# Tolerant Larvae and Sensitive Juveniles: Integrating Metabolomics and Whole-Organism Responses to Define Life-Stage Specific Sensitivity to Ocean Acidification in the American Lobster

**DOI:** 10.3390/metabo11090584

**Published:** 2021-08-30

**Authors:** Fanny Noisette, Piero Calosi, Diana Madeira, Mathilde Chemel, Kayla Menu-Courey, Sarah Piedalue, Helen Gurney-Smith, Dounia Daoud, Kumiko Azetsu-Scott

**Affiliations:** 1 Département de Biologie, Chimie et Géographie, Université du Québec à Rimouski, 300 allée des Ursulines, Rimouski, QC G5L 3A1, Canada; mathildechemel@yahoo.fr (M.C.); kmenucourey@gmail.com (K.M.-C.); sarahpiedalue@hotmail.com (S.P.); 2 ECOMARE-Laboratory for Innovation and Sustainability of Marine Biological Resources, CESAM-Centre for Environmental and Marine Studies, Department of Biology, University of Aveiro, Estrada do Porto de Pesca Costeira, 3830-565 Gafanha da Nazaré, Portugal; d.madeira@ua.pt; 3 Saint Andrews Biological Station, Fisheries and Oceans Canada, 125 Marine Science Drive, Saint Andrews, NB E5B 0E4, Canada; Helen.Gurney-Smith@dfo-mpo.gc.ca; 4 Homarus Inc., 408 rue Main, Shediac, NB E4P 2G1, Canada; dounia@mfu-upm.com; 5 EcoNov, 44 ave Bromley, Moncton, NB E1C 5T9, Canada; 6 Bedford Institute Oceanography, Fisheries and Oceans, Dartmouth, NS B2Y 4A2, Canada; Kumiko.Azetsu-Scott@dfo-mpo.gc.ca

**Keywords:** crustacean, early life stages, ontogeny, pH, *p*CO_2_, CCS, carryover effects, metabolomic reprogramming, global ocean change, oxygen consumption, feeding rate, mineralisation

## Abstract

Bentho-pelagic life cycles are the dominant reproductive strategy in marine invertebrates, providing great dispersal ability, access to different resources, and the opportunity to settle in suitable habitats upon the trigger of environmental cues at key developmental moments. However, free-dispersing larvae can be highly sensitive to environmental changes. Among these, the magnitude and the occurrence of elevated carbon dioxide (CO_2_) concentrations in oceanic habitats is predicted to exacerbate over the next decades, particularly in coastal areas, reaching levels beyond those historically experienced by most marine organisms. Here, we aimed to determine the sensitivity to elevated *p*CO_2_ of successive life stages of a marine invertebrate species with a bentho-pelagic life cycle, exposed continuously during its early ontogeny, whilst providing in-depth insights on their metabolic responses. We selected, as an ideal study species, the American lobster *Homarus americanus*, and investigated life history traits, whole-organism physiology, and metabolomic fingerprints from larval stage I to juvenile stage V exposed to different *p*CO_2_ levels. Current and future ocean acidification scenarios were tested, as well as extreme high *p*CO_2_/low pH conditions that are predicted to occur in coastal benthic habitats and with leakages from underwater carbon capture storage (CCS) sites. Larvae demonstrated greater tolerance to elevated *p*CO_2_, showing no significant changes in survival, developmental time, morphology, and mineralisation, although they underwent intense metabolomic reprogramming. Conversely, juveniles showed the inverse pattern, with a reduction in survival and an increase in development time at the highest *p*CO_2_ levels tested, with no indication of metabolomic reprogramming. Metabolomic sensitivity to elevated *p*CO_2_ increased until metamorphosis (between larval and juvenile stages) and decreased afterward, suggesting this transition as a metabolic keystone for marine invertebrates with complex life cycles.

## 1. Introduction

Bentho-pelagic life cycles are the dominant reproductive strategy in the oceans, as they are found in more than 75% of known marine invertebrate species [1]. This reproductive strategy can provide various benefits, including long distance dispersal, colonisation of new habitats, access to different resources, and competition avoidance of resources with conspecifics. However, pelagic early life stages are also subjected to increased mortality due to predation and exposure to environmental fluctuations experienced in their pelagic habitats [1]. Moreover, complex life cycles are punctuated by critical junctures along the ontogenetic trajectory, beginning at hatching when larvae are released in the water column, continuing with metamorphosis when larvae transform into post-larvae and reach the juvenile stages when they return to the sea floor [2]. These junctures represent bottlenecks, or biological filters, that reduce recruitment to the adult population [3].

Early life stages strongly differ from one another for their anatomical, physiological, and behavioural traits. Changes occurring throughout ontogeny progressively contribute to the ecological shift that takes place during the transition from the planktonic to the benthic habitat, with metamorphosis representing a fundamentally key moment during development [4,5]. Homeostatic and regulatory functions develop throughout ontogeny and, as a consequence, early life stages are overall considered to be more sensitive to environmental changes when compared to later life stages [6,7,8].

Along their ontogeny, with habitat transition, early life stages naturally face changes in temperature, salinity, dissolved oxygen (O_2_) and carbon dioxide (CO_2_), among other environmental drivers [6]. However, ongoing environmental changes are occurring at a faster rate than documented before, and are punctuated with more frequent and intense extreme events, when compared to those that organisms have experienced in their evolutionary history [9,10,11]. Among these changes, ocean acidification (OA) is recognised as a major threat for marine life, and in particular for calcifying species [12]. Due to the increase in atmospheric CO_2_ partial pressure (*p*CO_2_), pH in surface waters is predicted to decline by 0.3 pH units by the end of the century, according to the Intergovernmental Panel on Climate Change (IPCC RCP 8.5; [13]). Modifications of seawater carbonate chemistry due to OA also lead to a decrease in carbonate ion concentrations (CO_3_^2−^) [14] and a reduction in the calcium carbonate saturation state (Ω), which regulates the thermodynamics of calcium carbonate (CaCO_3_) precipitation [15]. However, pH levels predicted to occur by the end of the century for the open ocean have already been reached in coastal areas, and even lower levels have already been documented, associated with upwellings, freshwater influxes, and eutrophication events [16,17]. These pH decreases, and changes in carbonate chemistry, are known to affect marine organisms’ mineralisation and mineralised structures [18]. In addition, these conditions challenge a number of marine organisms physiologically, more specifically impacting their energy metabolism, acid-base balance, osmo-ionic regulation, and general homeostasis [19]. Whether and how marine invertebrates’ early life stages will be able to cope with these environmental changes is still poorly understood [20], particularly from a metabolic perspective.

Among marine invertebrates, crustaceans, and especially decapods, are considered to be among the least affected calcifying species by the exposure to low pH/high *p*CO_2_ conditions [8,12] in terms of survival, growth, metabolism, acid-base balance, carapace mineralisation, and behaviour [21,22,23,24,25,26]. While different species can show opposite responses [27,28], this overall low sensitivity to OA may be linked to crustaceans homeostatic and regulatory abilities, their capacity to relocate exoskeletal calcium (Ca^2+^) and carbonate (CO_3_^2−^) ions during moulting, and their impermeable cuticle protecting the carapace from dissolution and limiting exchanges with the surrounding environment [28,29,30]. Results of studies on decapods’ sensitivity to OA are disparate, especially considering the discrepancy in results across species and life stages (e.g., [27,31,32,33,34,35]). In addition, most studies on this subject have mainly focused on life history and developmental traits [8], creating an important paucity in terms of our understanding of the metabolic mechanisms involved in OA responses [36]. This is also true for species of great economic importance, such as the American lobster (*Homarus gammarus*, Milne Edwards, 1837), which supports the most valuable and lucrative single fishery in Atlantic North America [37,38].

Lobster pre-larvae, hatched from the egg mass carried by the female, moult into the first mobile zoea larval stage (stage I) [4]. This stage is followed by two other planktonic zoea stages (stage II and III), each separated from one another by a moult. Subsequently, a metamorphic moult occurs at the end of stage III, leading to the post-larval stage IV. Stage IV lobsters eventually reach the seafloor to search for a suitable habitat in which to settle, swimming between the water column and the benthic habitat. When they finally settle, another moult transforms post-larvae into stage V juveniles. A series of juvenile stages follows, ultimately leading to the adult stage. Along with ontogeny, different studies have shown that OA conditions could affect survival, development, growth, carapace composition and size, feeding capacities, swimming behaviour, and oxygen consumption, but also elementary composition and gene expression in some stages [36,39,40,41,42].

While these studies have a great value in outlining the sensitivity of the American lobster to OA, improving our appreciation of the metabolic challenges that early life stages face under OA conditions requires: (i) investigating multiple successive early life stages, not excluding developmental keystones; (ii) determining early life stages responses to a discreet pH/*p*CO_2_ gradient, instead of single or few specific OA scenarios, which enables us to produce more robust predictions of physiological responses and supports science-informed management decisions [43,44,45,46]; and (iii) explicitly investigating the metabolic implications of exposure to low pH/high *p*CO_2_ [47]. Consequently, the aim of our work was to simultaneously investigate life history traits and metabolic (both at the cellular and whole-organism level) responses of successive early life stages exposed to a range of elevated *p*CO_2_ conditions.

We exposed consecutive stages I to V of the American lobster to seven different *p*CO_2_ representing current (400 µatm), OA predictions for open ocean (600, 800, and 1000 µatm), and extreme levels which are already experienced in coastal benthic environment, and are expected to occur more frequently with predicted global changes and potential leakages from underwater carbon capture storages (CCS) (1200, 2000, and 3000 µatm). We particularly investigated changes in survival, developmental time, morphometrics, mineralisation, metabolic rates, and metabolic pathway utilisation as a function of seawater *p*CO_2_ in early development. As strong changes in anatomy, physiology, ecology, and behaviour occur during development, we hypothesized that progressive changes in metabolic functions and sensitivity to elevated *p*CO_2_ will occur with ontogeny. In line with the historical view that larvae are less tolerant to environmental changes [6,48], we hypothesized on one hand that larval stages would be more sensitive than juveniles to high *p*CO_2_ especially due to their poor homeostatic and regulatory functions. On another hand, the ongoing organogenesis and progressive mineralisation of the carapace of juvenile stages would cause them to be more negatively impacted by elevated *p*CO_2_ than larvae, according to a more modern view [34,42]. Finally, given the significance of metamorphosis occurring between stage III and stage IV [4], we specifically hypothesized that the sensitivity to elevated *p*CO_2_ is highest at this transition phase between the last larval and post-larval/first juvenile stage, particularly for the metabolic functions investigated.

## 2. Results

### 2.1. Functional Changes across Ontogeny

Throughout their development, early life stages of the American lobster *Homarus americanus* changed significantly, not only in terms of body size and carapace mineralisation (main components Ca^2+^, Mg^2+^, Na^+^, K^+^, and Sr ^+^), but also in terms of metabolic rates. Fundamentally, all traits increase from one stage to the next along lobsters’ ontogenetic trajectory (Tables S1–S3). Briefly, for body size, from stage I to stage V (i) mean body size increased progressively from 4.72 ± 0.05 to 9.38 ± 0.12 mm, (ii) mean cephalothorax length increased from 1.93 ± 0.03 to 4.57 ± 0.06 mm, and (iii) mean abdomen size increased from 2.78 ± 0.03 to 4.80 ± 0.07 mm (Table S1). In addition, stage IV and V lobsters show higher carapace [Ca^2+^] and [Mg^2+^] and lower carapace [K^+^] when compared to the larval stages (Table 1 and Table S2). In particular, [Ca^2+^] showed a major increase between stage III and stage IV (Table S2).

**Figure 1:**
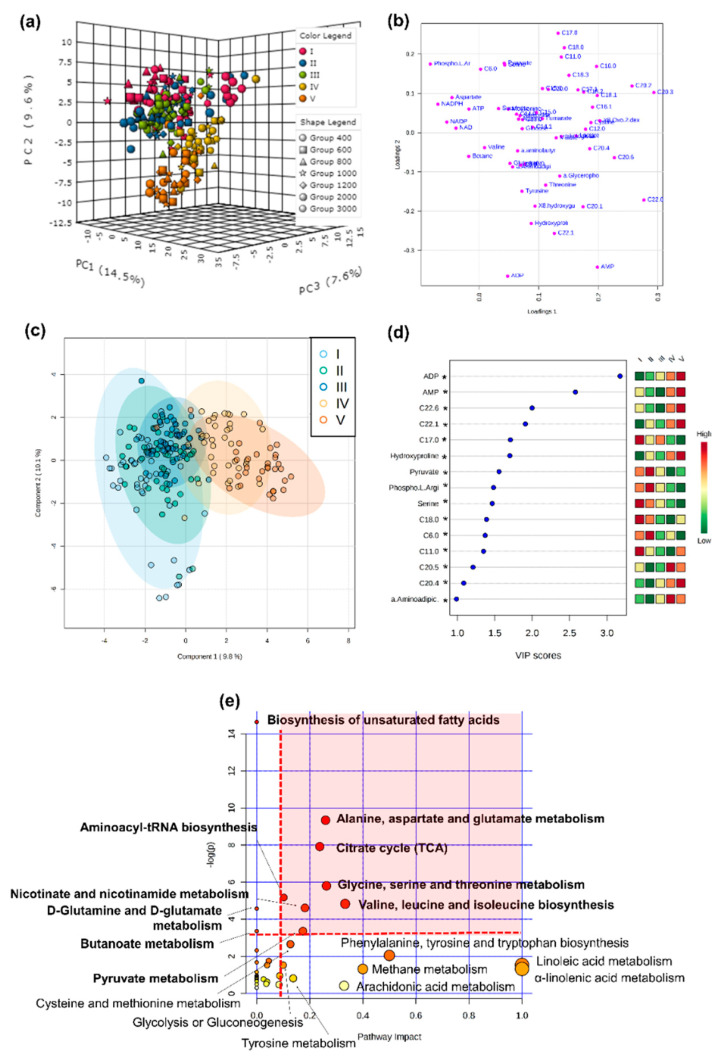
**Metabolome differences between developmental stages of the American lobster *Homarus americanus*.** (**a**) PCA 3D score plot, (**b**) PCA 2D loadings plot, (**c**) PLS−DA score plot with 95% confidence intervals. Model validation was calculated by the prediction accuracy during training test statistics with 1000 permutations, *p* = 0.001, (**d**) PLS−DA Variable Importance in Projection scores (VIP > 1 for significance). Metabolites with an asterisk were also significant in the ANOVA, with *p* < 0.05 (for full ANOVA results see Table S5). The coloured boxes on the right indicate the relative concentrations of the corresponding metabolite in each developmental stage, (**e**) pathway analysis of metabolomic changes carried out with the statistically significant metabolites (ANOVA *p* < 0.05). Hypergeometric test was used for over-representation analysis (*p* < 0.05 for significance) and relative betweenness centrality for pathway topology analysis (impact > 0.1). Annotated pathways were important in developmental processes based on either criterion. Bold pathways indicate *p* < 0.05. Pathways within the red area were considered the most relevant as they have *p* < 0.05 and impact > 0.1.

Metabolomic profiles differed significantly between larval and post-metamorphosis stages, as the metabolomes of developmental stages formed two discernible clusters for the different phases (Figure 1). More specifically, one grouping included stage I, II, and III, and the other grouping stage IV and V (explained variance of 24.1% for PC1 and PC2, Figure 1a), despite a progressive shift from stage I to stage V metabolomics profile being evident (Figure 1c). Metabolite C17:0 presented higher concentrations during early developmental stages, decreasing toward post-metamorphosis stages. ADP, AMP, C22:6, and C22:1 were also among the top five ranked metabolites with greater discriminatory power, which mostly increased in later developmental stages (Figure 1d). The full list of metabolites that significantly changed throughout ontogeny are involved in 18 pathways, identified as important in developmental processes (red area, Figure 1e).

### 2.2. Stage-Specific pCO_2_ Effect on Survival Rates

**Figure 2:**
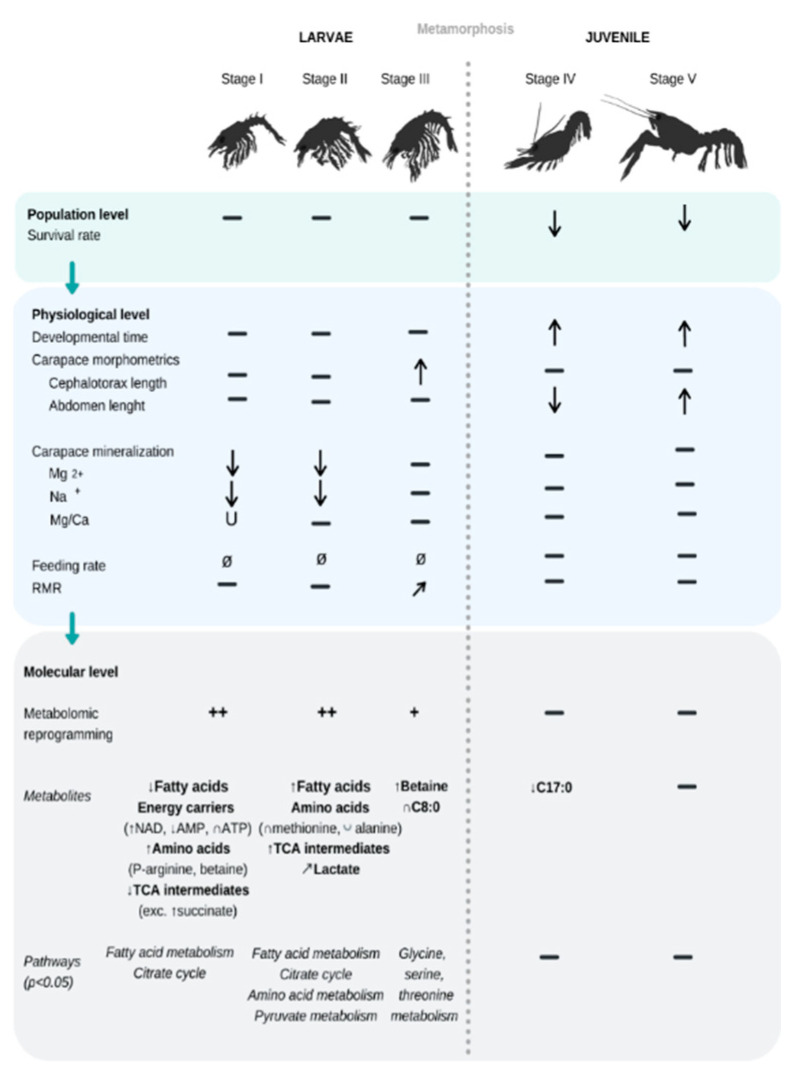
Summary of the significant effects of increasing *p*CO_2_ conditions (from 400 to 3000 µatm, i.e., pH 8.0 to 7.2) on the biological traits measured in the present study, for five consecutive early life-stages of the American lobster *H. americanus* (from larval stage I to juvenile stage V). Arrows and U indicate the direction of the response to increasing *p*CO_2_—means no change detected with *p*CO_2_, while + indicates a semi-quantitative intensity of reprogramming. Ø is for a trait that was not measured for certain life stages. No feeding rate was measured on larvae.

**Figure 3:**
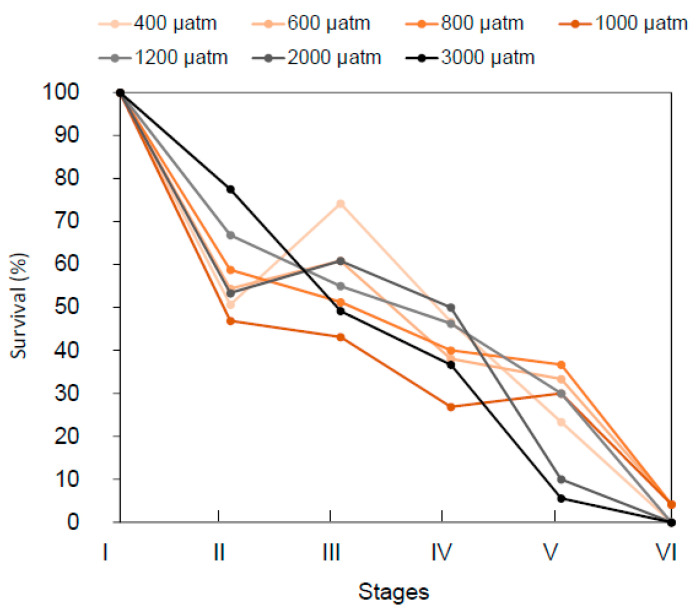
**Survival**. Survival rates as percentages of the initial larvae concentration (600 larvae per tank) for five early life stages of the American lobster *H. americanus* exposed to *p*CO_2_ conditions mimicking the current *p*CO_2_ condition, ocean acidification scenarios or during upwellings (600, 800, 1000, 1200, and 2000 µatm) and carbon capture storage leaks (3000 µatm). Dots represent the mean value (*n* = 3 to 4 tank replicates per *p*CO_2_ condition), SE bars are not represented for a better legibility.

Cumulative survival rates were negatively impacted by increasing *p*CO_2_ (Figure 2 and Figure 3), whilst survival between stages (Table S1) was differentially affected by *p*CO_2_ over developmental stages, as indicated by the presence of a significant interaction between the terms “*p*CO_2_” and “Stage” (Table 1). Specifically, a 42% survival decrease was observed between stage I and II for all the *p*CO_2_ conditions, without any clear pattern of differences as a function of *p*CO_2_. At the transition between stage III and IV, survival rates were between 30 and 50%, with the best survival rates recorded for *p*CO_2_ conditions of 1200 and 2000 µatm. At stage V, the survival drastically dropped to 10% for the two highest *p*CO_2_ conditions, and the highest survival rates (33 and 37%) were recorded at *p*CO_2_ 600 and 800 µatm, respectively. Finally, at the juvenile stage VI, less than 5% of the initial number of larvae survived at 600 and 1000 µatm *p*CO_2_, whilst no survivor was recorded for all other treatments. Comparing the specific survival at each stage showed that only stage V survival significantly decreased with the increasing *p*CO_2_ (linear model, *p* = 0.003).

### 2.3. Stage-Specific pCO_2_ Effect on Developmental Time

**Figure 4:**
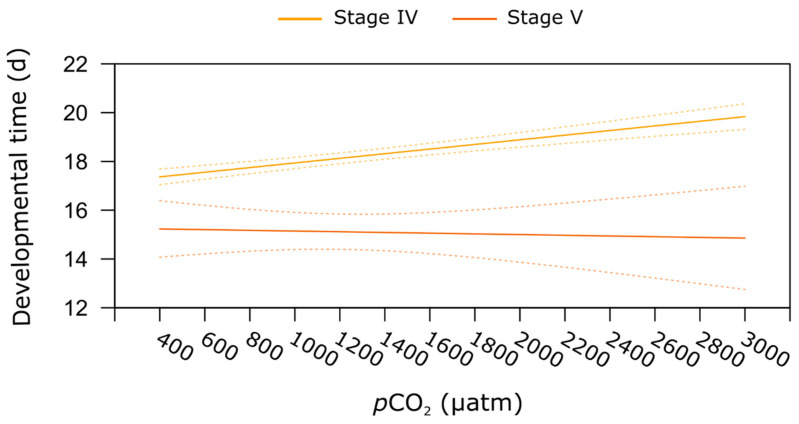
**Juvenile stages developmental time**. Relationship between seawater *p*CO_2_ and developmental time (i.e., period between two moults) in stage IV post-larvae and stage V juveniles of the American lobster *H. americanus* exposed to a *p*CO_2_ gradient. Full curves represent the predicted values (from the linear model) for developmental time as a function of *p*CO_2_ for each stage; dashed lines represent the 95% upper and lower confidence intervals.

Elevated *p*CO_2_ did not affect the duration of the development (i.e., developmental time) in the three larval stages. Transition between stages lasted on average 5 d for stages I to II and stages III to IV, and 6 d between stages II and III. As we used different techniques to determine developmental time (in batch for larvae versus individually for juveniles), the interaction between all life stages and *p*CO_2_ was not tested. However, for juveniles, exposure to increasing *p*CO_2_ significantly extended the duration of the developmental period, regardless of the post-larvae and juvenile stages (linear model, *p* < 0.001, Table 1). For stage IV, the developmental period increased significantly from 18 d at 400 µatm to 21 d at 3000 µatm of *p*CO_2_. The mean duration of the development period for stage V was 15 d, therefore shorter than for stage IV juveniles (Table 1, stage effect *p* < 0.001). Finally, we observed an increase in variation of the mean of the duration for the developmental period at the highest *p*CO_2_ tested, this pattern being more marked at stage V when compared to stage IV (see confidence interval, Figure 4).

### 2.4. Stage-Specific pCO_2_ Effect on Carapace Morphometrics

**Figure 5:**
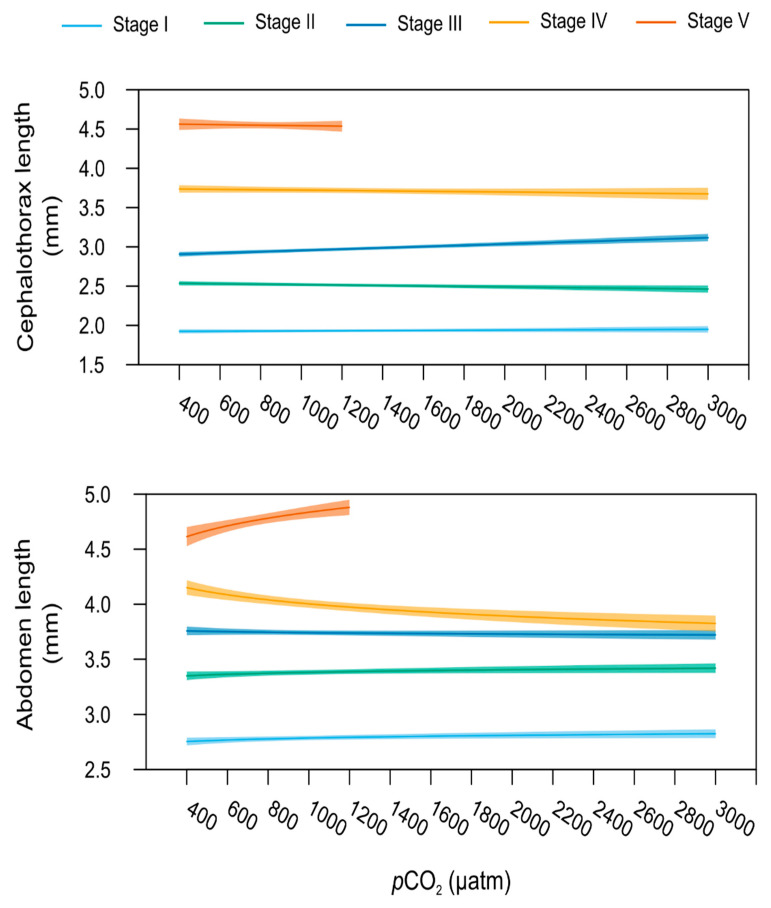
**Morphometrics**. Relationship between the cephalothorax and abdomen lengths (in mm) and the seawater *p*CO_2_ for the early life stages of the American lobster. Bold lines represent the predicted values (from the linear and logarithmic model for cephalothorax and abdomen, respectively) as a function of *p*CO_2_ for stage I (light blue), stage II (green), stage III (dark blue), stage IV (yellow), and stage V (orange), with the total length (covariate) fixed at the stage mean value for each stage. Shaded areas around regression lines represent the 95% confidence interval of the predicted values. Models stop at 1200 µatm for stage V juvenile lobsters, as all the individuals were dead in the 2000 and 3000 µtm treatments.

Lobsters’ cephalothorax, abdomen, and tail length varied in relation to increasing levels of *p*CO_2_ depending on the life stage, as indicated by the presence of a significant interaction between the terms “*p*CO_2_”and “Stage” for all these variables (linear models, Table 1). *p*CO_2_-dependent variation of cephalothorax length was best explained by a linear relationship, whilst abdomen and tail lengths by a logarithmic relationship (Table S4). In more detail, larval stage I and II morphometric measurements were comparable across all the different *p*CO_2_ conditions with a difference of 16 and 9% between the shortest and the longest cephalothorax and abdomen recorded, respectively (Figure 5). As a result, exposure to increasing seawater *p*CO_2_ did not affect these traits in larval stages I and II (stage-specific linear model, *p* > 0.05). Conversely, cephalothorax length of stage III larvae increased significantly with increasing seawater *p*CO_2_ (stage-specific linear model, *p* < 0.001). Mean cephalothorax length ranged from 2.78 to 3.15 mm, respectively measured at 400 to 3000 µatm, i.e., a 36% increase in this trait. Finally, no effect of seawater *p*CO_2_ was found on stage III abdomen length (stage-specific linear model, *p* > 0.05). Juvenile stages showed opposite responses when compared to those observed at stage III, with a slight decrease in abdomen length with increasing *p*CO_2_ (stage-specific linear models, *p* = 0.019 for stage IV and *p* = 0.004 for stage V; Figure 5). However, seawater *p*CO_2_ had no significant effect on mean cephalothorax length at stage IV and V.

In general, increasing *p*CO_2_ alone did not affect morphometric parameters except for the total length of individuals (linear model, all stages included, *p* < 0.001; Table 1), which tended to increase slightly with increasing *p*CO_2_ (Table S1). Total length measured at 400 µatm was generally the shortest length, and the increase in *p*CO_2_ led to an increase in length of 10 to 20% from one stage to another.

### 2.5. Stage-Specific pCO_2_ Effect on Carapace Mineralisation

Among the six ions measured in the lobsters’ carapace, only [Mg^2+^] and [Na^+^] varied significantly with increasing levels of *p*CO_2_ depending on the life stage (linear model, *p* = 0.033 and 0.034, respectively, Table 1). [Mg^2+^] and [Na^+^] *p*CO_2_-dependent responses were best explained by a linear relationship (Table S4), whilst a polynomial model better fitted [Mg^2+^]:[Ca^2+^]-*p*CO_2_ relationship (model polynomial 2nd degree, Table 1 and Table S4).

**Figure 6:**
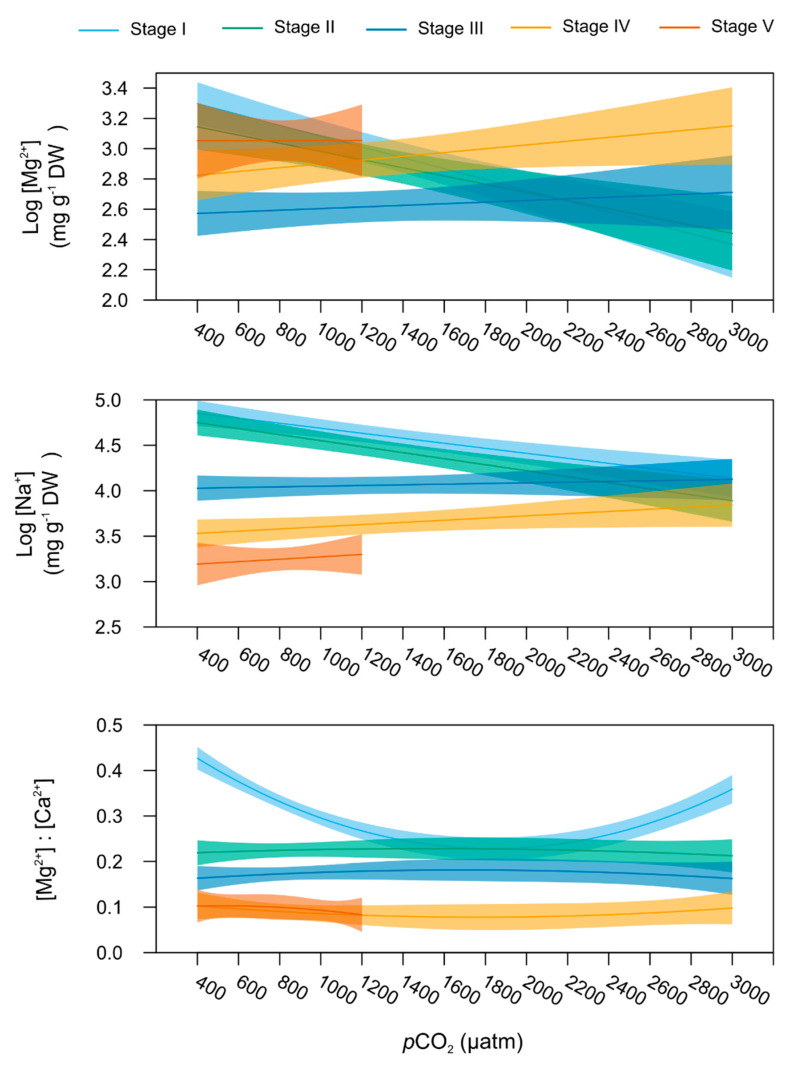
**Mineralisation**. Relationship between mineralisation contents and the seawater *p*CO_2_ for the early life stages of the American lobster. Bold lines represent the predicted values (from the linear for Mg^2+^ and Na^+^ contents and polynomial model for [Mg]:[Ca] ratio as a function of *p*CO_2_ for stage I (light blue), stage II (green), stage III (dark blue), stage IV (yellow), and stage V (orange), with the total wet body mass (covariate) fixed at the stage mean value for each stage. Shaded areas around the lines represent the 95% confidence interval of the predicted values. Models stop at 1200 µatm for stage V juvenile lobsters, as all the individuals were dead in the 2000 and 3000 µtm treatments.

In particular, larvae stage I and II (Figure 6) were seen to possess lowering carapace [Mg^2+^] and [Na^+^] with seawater *p*CO_2_ increasing from 400 to 3000 µatm (stage-specific linear models, stage I: *p_Mg2+_* = 0.004 and *p_Na+_* = 0.026, and stage II: *p_Mg2+_* = 0.045 and *p_Na+_* = 0.006). No significant *p*CO_2_ effect was observed on these mineral contents for larvae stage III and subsequent juvenile stages (stage-specific linear models, *p* > 0.05).

In stage I larvae, [Mg^2+^]:[Ca^2+^] described a U-shaped response pattern to increasing *p*CO_2_ (stage-specific linear model, *p* = 0.04). The lowest ratios were observed at 800 and 1000 µatm (Figure 6). Even if none of the stage-specific linear model showed a significant *p*CO_2_ effect, stages II and III showed the highest [Mg^2+^]:[Ca^2+^] values for *p*CO_2_ between 800 and 1200 µatm (Figure 5, Table S2) while stage IV presented the lowest [Mg^2+^]:[Ca^2+^] ratio at 600 and 800 µatm (Table S2). Finally, mean cation ratios did not change significantly across the *p*CO_2_ gradient for stage V lobsters.

Carapace [K^+^] was the only parameter not showing stage-specific sensitivity to seawater *p*CO_2_, as it followed a comparable slight linear decreasing trend with increasing seawater *p*CO_2_ across all stages (*p* = 0.033, Table 1).

### 2.6. Stage-Specific pCO_2_ Effect on Resting Metabolic and Feeding Rates

**Figure 7:**
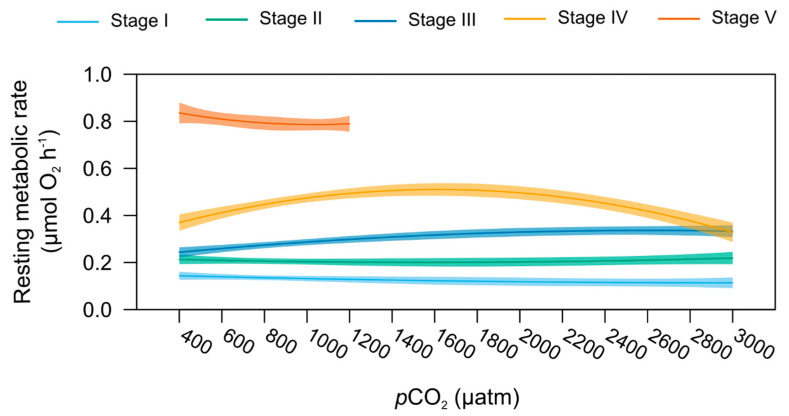
**Resting metabolism.** Relationship between the resting metabolic rates (in µmol O_2_ h^−1^) and seawater *p*CO_2_ for the early life stages of the American lobster. Bold lines represent the predicted values (from the polynomial model) in function of *p*CO_2_ per stage, with the wet body mass (covariate) fixed at its mean value for each stage, and temperature (second covariate) set at 18 °C. Shaded areas around regression lines represent the 95% confidence interval of the predicted values. The model stops at 1200 µatm for stage V juvenile lobsters, as there were not enough individuals at 2000 and 3000 µtm to carry out respirometry.

Resting metabolic rates (MO_2_) responded differently to increasing seawater *p*CO_2_ at the different life stages investigated, as shown by the presence of a significant interaction between the term “Stage” and “*p*CO_2_” (polynomial model, *p* < 0.001, Figure 2). However, no significant *p*CO_2_ effect on MO_2_ was detected using stage-specific models (Figure 7). It is noted that stage III MO_2_ trended upwards with the increasing *p*CO_2_ from 0.17 µmol O_2_ h^−1^ at 400 µatm to 0.30 µmol O_2_ h^−1^ at 3000 µatm (Table S3).

The increase in *p*CO_2_ did not significantly affect feeding rate, neither in juvenile stage IV (mean ± SE = 9.51 ± 1.18 mg fish h^−1^ all *p*CO_2_ considered) nor in juvenile stage V (mean ± SE = 11.44 ± 1.23 mg fish h^−1^ all *p*CO_2_ considered, Table 1).

### 2.7. Stage-Specific pCO_2_ Effect on Metabolomic Fingerprints

**Figure 8:**
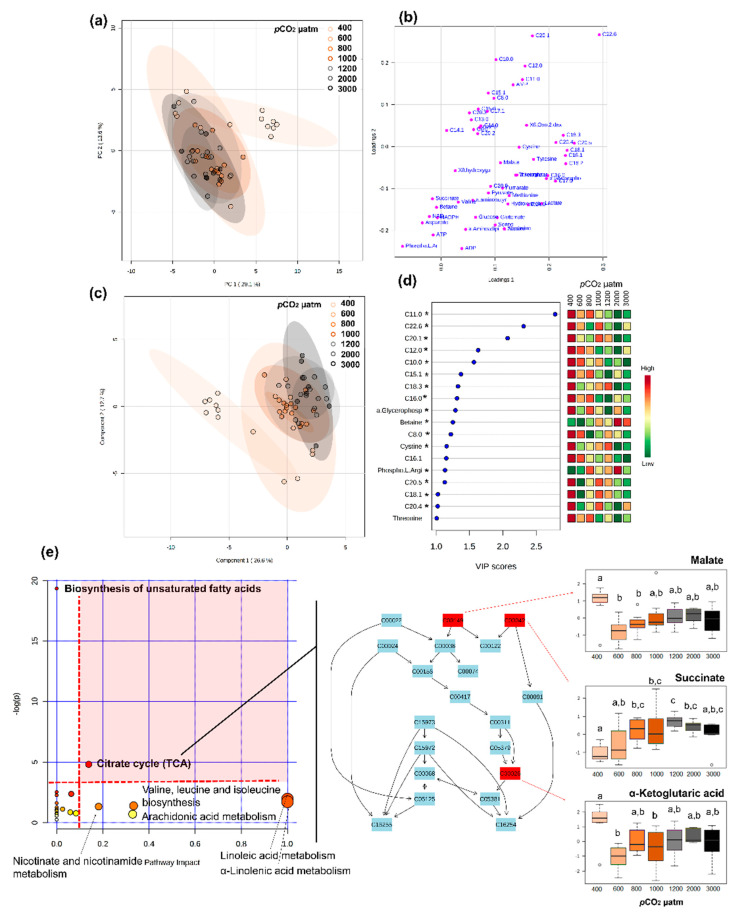
**Metabolomic fingerprinting larvae stage I.** Metabolome differences between *p*CO_2_ treatments in lobster larval stage I (**a**) PCA 2D score plot with 95% confidence intervals, (**b**) PCA 2D loadings plot, (**c**) PLS-DA score plot with 95% confidence intervals. Model validation was calculated by the prediction accuracy during training test statistics with 1000 permutations, *p* = 0.028, (**d**) PLS-DA Variable Importance in Projection scores (VIP > 1 for significance). Metabolites with an asterisk were also significant in the ANOVA, with *p* < 0.05 (for full ANOVA results see Table S5). The coloured boxes on the right indicate the relative concentrations of the corresponding metabolite in each *p*CO_2_ treatment: and (**e**) pathway analysis of metabolic changes carried out with the statistically significant metabolites (ANOVA *p* < 0.05). Hypergeometric test was used for over-representation analysis (*p* < 0.05 for significance, pathways highlighted in bold) and relative betweenness centrality for pathway topology analysis (impact > 0.1 for relevance). Annotated pathways were important in response to *p*CO_2_ based on either criterion. Pathways within the red area were considered the most relevant as they have *p* < 0.05 and impact > 0.1, i.e., citrate cycle. On the right panel, the citrate cycle is represented, with metabolites coded by KEGG number; metabolites coloured in red varied significantly. Boxplots of each compound highlighted in red were constructed based on the 25th and 75th percentiles (bottom and top of the box) with the middle line representing the median. The upper whisker represents the smaller maximum value and Q3 + 1.5 × IQR (Interquartile Range) and the lower whisker represents the larger minimum value and Q1 − 1.5 × IQR. Different letters indicate significant differences (*p* < 0.05) between groups.

**Figure 9:**
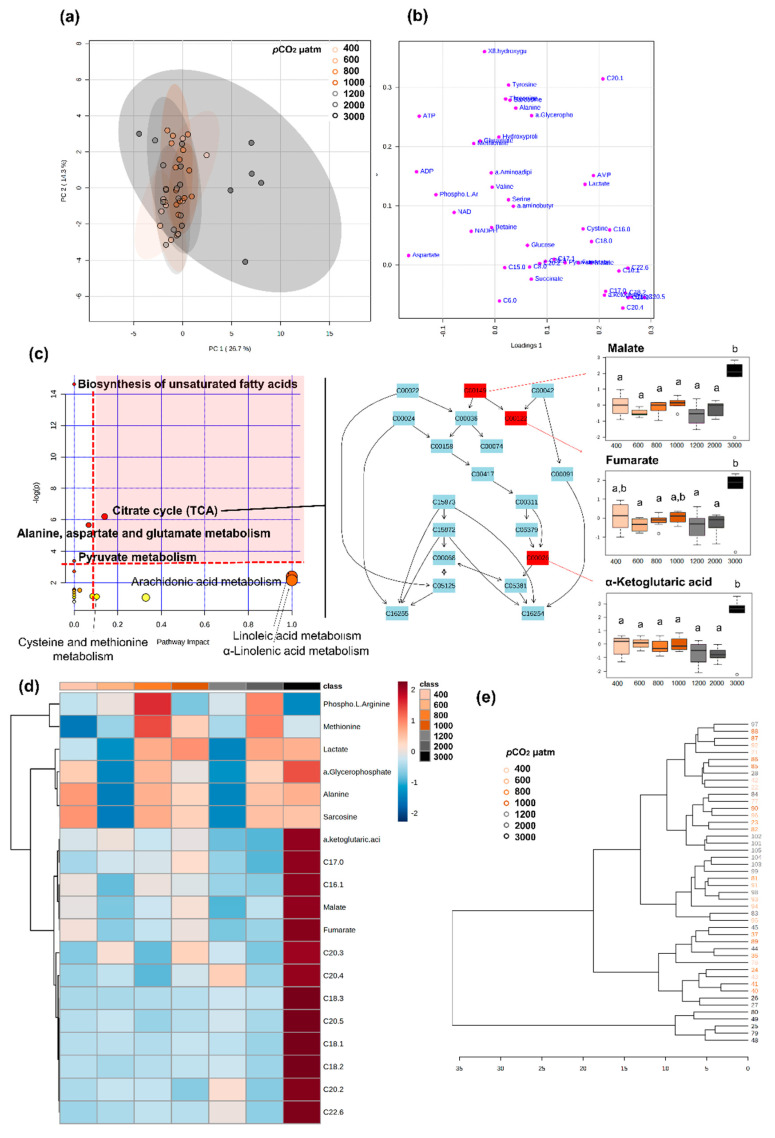
**Metabolomic fingerprinting larvae stage II**. Metabolome differences between *p*CO_2_ treatments in lobster larval stage II (**a**) PCA 2D score plot, (**b**) PCA 2D loadings plot, (**c**) pathway analysis of metabolic changes carried out with the statistically significant metabolites (ANOVA *p* < 0.05). Hypergeometric test was used for over-representation analysis (*p* < 0.05 for significance, pathways highlighted in bold) and relative betweenness centrality for pathway topology analysis (impact > 0.1 for relevance). Annotated pathways were important in response to *p*CO_2_ based on either criterion. Pathways within the red area were considered the most relevant, as they have *p* < 0.05 and impact > 0.1, i.e., citrate cycle. On the right panel, the citrate cycle is represented, with metabolites coded by KEGG number; metabolites coloured in red varied significantly. Boxplots of each compound highlighted in red were constructed based on the 25th and 75th percentiles (bottom and top of the box) with the middle line representing the median. Upper whisker represents the smaller maximum value and Q3 + 1.5 × IQR (Interquartile Range) and the lower whisker represents the larger minimum value and Q1 − 1.5 × IQR. Malate, fumarate, and α-ketoglutaric acid measured at 3000 µatm *p*CO_2_ were significantly different (as indicated by different letters, *p* < 0.05) from the other *p*CO_2_ treatments, (**d**) Heat map of clustered data (distance measure: Pearson correlation, clustering algorithm: complete linkage) in which cells denote the log transformed and pareto-scaled values of metabolite concentration. The colour scale ranges from red (higher than mean concentration) to blue (lower than mean concentration). Rows are the metabolites that were significantly different between treatments (ANOVA, *p* < 0.05) and columns are the *p*CO_2_ treatments, and (**e**) Hierarchical clustering of samples (distance measure: Euclidean distance, clustering algorithm: Ward) to unravel data structure. *p*CO_2_ treatments.

Metabolomics profiles of larval stages I and II differed significantly when exposed to different seawater *p*CO_2_ conditions (Figure 8 and Figure 9, respectively).

For stage I, 18 metabolites showed VIP scores > 1, the top five being fatty acids (C11:0, C22:6, C20:1, C12:0, and C10:0), which mostly decreased with increasing *p*CO_2_ (Figure 8). Thirty-two metabolites were found to change significantly among *p*CO_2_ treatments, including lipids, energy carriers, amino acids, and a marker of DNA oxidation (Table S5). Pathway analysis showed that these metabolites were involved in seven pathways (considered relevant according to either over-representation *p* < 0.05 or pathway topology analysis with impact > 0.1), including: biosynthesis of unsaturated fatty acids, citrate cycle, nicotinate and nicotinamide metabolism, amino acid metabolism and fatty acid metabolism (Figure 8e). Among these, the most relevant pathway (combination of over-representation *p* < 0.05 and pathway impact > 0.1) was the citrate cycle (Figure 8e, Table S5). Malate and α-ketoglutaric acid decreased with increasing seawater *p*CO_2_ and succinate mostly increased with increasing *p*CO_2_.

For stage II larvae, group separation was not very clear in the PCA, as there is some overlapping between treatments, even though larvae exposed to 3000 µatm seem to respond differently (Figure 9). Moreover, no conclusion could be reached about which metabolites had discriminative power, as the PLS-DA model was not significant. Nonetheless nineteen metabolites were found to change significantly between seawater *p*CO_2_ treatments, including lipids, citrate cycle metabolites, lactate, and amino acids (Table S6). Pathway analysis showed that these metabolites were involved in eight relevant pathways (according to either over-representation *p* < 0.05 or pathway topology analysis with impact > 0.1) including biosynthesis of unsaturated fatty acids, citrate cycle, pyruvate metabolism, amino acid metabolism, and fatty acid metabolism (Figure 9c,d). Among these, the most relevant pathway (combination of over-representation *p* < 0.05 and pathway impact > 0.1) was the citrate cycle, similarly to stage I larvae (Figure 8c, Table S6). Conversely, in this case, malate, fumarate, and α-ketoglutaric acid increased in larvae exposed to 3000 µatm of *p*CO_2_, remaining low in the other *p*CO_2_ treatments (Figure 9a,b).

Stage III, IV, and V lobsters did not undergo extensive metabolic reprogramming when exposed to increasing *p*CO_2_/decreasing pH, compared to larval stages I and II. For these three stages, different *p*CO_2_ treatments show a high degree of overlapping (Figure S1), and PLS-DAs showed no significant pattern (prediction accuracy during training test statistic with 1000 permutations, *p* > 0.05).

Stage III larvae showed very few significant differences in metabolome when exposed to different seawater *p*CO_2_. Betaine increased at 600, 800, 1200, and 3000 µatm seawater *p*CO_2_, when compared to control conditions (400 µatm), and C8:0 increased significantly at 1200 µatm in relation to control. Despite the low number of significant metabolites, the pathway analysis showed one significant pathway, which was glycine, serine, and threonine metabolism (over-representation analysis, *p* = 0.045; pathway topology, impact = 0.0).

Similarly, only C17:0 varied significantly in stage IV lobsters, decreasing as *p*CO_2_ rose (ANOVA, F = 5.1, *p* = 0.001). No significantly changed pathway was detected in stage IV lobsters exposed to increasing seawater *p*CO_2_. Finally, there were no metabolome differences between stage V lobsters exposed to different *p*CO_2_ conditions (ANOVA, *p* > 0.05 for all metabolites, Figure S1).

## 3. Discussion

Our study comprehensively integrates the investigation of life history, mineralisation, and metabolic and metabolomic responses to a broad seawater *p*CO_2_ gradient across multiple early ontogenetic stages, in an ecological and economic important marine invertebrate species with a bentho-pelagic life cycle, the American lobster *Homarus americanus*. As such, our work represents the largest most comprehensive study of its kind. In addition, and most importantly, the complementarity of the tools used, and the biological variables measured, provide compelling evidence that metabolomic reprogramming at the cellular level buffers, at the whole organism level, the physiological and morphological responses to the exposure to elevated seawater *p*CO_2_.

Here, we document the metabolic reprogramming that lobster early life-stages undergo throughout their development, and then focus on the main objective of this study by discussing stage-specific *p*CO_2_ responses separately for stage I–II larvae, stage III pre-metamorphosis, and post-metamorphosis stage IV–V. We give particular consideration to the integration of both whole-organism and cellular responses to elevated seawater *p*CO_2_ across development in an innovative approach, to gain a better, more comprehensive, and thorough understanding of lobster early life stage capacities to face OA conditions.

### 3.1. Metabolomic Reprogramming throughout Development in the American Lobster

An ontogenetic change in metabolomic signatures is evident in *H. americanus*, with a turning point corresponding to metamorphosis happening between stages III and IV. Pathways that change during development are mostly related to energy regulation, namely amino acid synthesis and metabolism, citrate cycle, aminoacyl-tRNA biosynthesis, and unsaturated fatty acids biosynthesis. This finding is supported by the presence of shifts in the concentration of metabolites such as ADP, AMP, and unsaturated fatty acids, which increase in post-metamorphic lobsters; while some saturated fatty acids, pyruvate and amino acids are more concentrated in pre-metamorphic lobsters. Metamorphosis thus seems to induce a metabolic shift from an amino-acid based metabolism to an unsaturated fatty acid-based metabolism, although a change in diet may also partly explain the observed changes. Studies on spiny lobsters suggest that the final larval instar needs to reach a critical and specific level of energy storage to support the swimming activity of the non-feeding puerulus (i.e., the intermediary free-swimming nektonic phase between pelagic larvae and benthic juveniles) and its subsequent moult to the first juvenile stage, with lipids being crucial for metamorphosis progression [49]. This could potentially explain the transition to an unsaturated-fatty acid-based metabolism upon metamorphosis in lobsters, as fatty acids and phospholipids are important sources of energy for lobster growth [49,50]. We further propose that the transition in metabolome signatures may be related to the development of specific organs (and thus new functions, e.g., osmo-ionic regulatory control), as amino acids need to be used as building blocks for biomass accumulation. For example, ontogenetic metabolomic profiling in the zebrafish *Danio rerio* (Hamilton, 1822) revealed the importance of nucleotides and glucose during early embryogenesis, while amino acids and intermediates of the citrate cycle were highly abundant in later stages, coinciding with the organogenesis of the liver and pancreas [51]. Hence, a similar trend could occur in invertebrate species, as tissue building blocks are conserved across the animal kingdom.

### 3.2. Larval Stages I and II: The Establishment of the Internal Regulation Machinery

Pelagic larval stages are considered particularly sensitive to environmental fluctuations [1,52]. In this sense, larval stages have been shown to be negatively affected by OA [53]. In particular, the first larval stage of the red king crab, *Paralithodes camtschaticus* (Tilesius, 1815) [54] and the European lobster *Homarus gammarus* (Linneaus, 1758) [33,34] exposed to OA conditions showed drastic decrease in survival rates, with these stages being considered as bottlenecks (or biological filters) for the recruitment to the adult populations [35]. Conversely, the survival of stage I and II larvae is not impacted by exposure to OA conditions in our study. Similar results were shown by Waller et al. [42] in the American lobster, supporting the idea that first larval stages of this species are highly tolerant to elevated *p*CO_2_, as it is for other crustaceans regardless of female pre-exposure to high *p*CO_2_ [55,56,57].

The duration of the larval development was similar in all the *p*CO_2_ conditions, conversely to the results reported by Keppel et al. [39]. In fact, the intermoult period between larval stages lasts approximately 5–6 d, matching the values measured for the same species at 19 °C [42]. As already shown for the European [34] and the American [42] lobsters, the increase in seawater *p*CO_2_ does not seem to cause an increase in the duration of the larva intermoult period in our study. This again supports the idea that larvae are highly tolerant to increasing seawater *p*CO_2_, even a drastic increase leading to pH values of 7.4, expected during coastal acidification events [17].

Similarly, zoea larvae morphometrics are not affected by exposure to OA in stages I and II; the mean carapace length compares broadly to values reported for the same species by Waller et al. [42]. This differs from the *p*CO_2_-dependent decrease in carapace length in American lobsters’ stage II and III individuals reported previously [39]. Differences between Keppel et al. [39] and our study might be due to differences in culturing conditions: notably their elevated temperature (20 °C), which also induced delayed moulting under OA conditions. Equally different starting conditions and environmental history of individuals brought to the laboratory may play an important role.

Low pH conditions which are induced by OA or other phenomena such as upwelling can challenge mineralised structures (such as carapaces and shells) and mineralisation, the former being decreases in calcium carbonate saturation state [15], and the latter demanding increased energy investment to replace damaged carbonated structures. In American lobster stage I and II larvae, we report a linear decrease in [Mg^2+^] and [Na^+^] along the *p*CO_2_ gradient tested. The decrease in [Mg^2+^] was also observed for the larvae of the European lobster [34]. In calcified structures, this decrease in [Mg^2+^] is usually concomitant with a reduction in [Ca^2+^] caused by a reduction in mineralisation and an increase in dissolution (e.g., [54,58]). However, we observe no reduction in [Ca^2+^] in larval stages of the American lobster, as reported for the European lobster [34]. Lobster larvae are poorly calcified [32], and we were not able to separate the carapace from the flesh for mineralisation analyses in larval stages. Thus, changes in [Mg^2+^] and [Na^+^] observed may be related to changes in carapace precipitation/dissolution, but in part (although we consider it marginal) also to energetic trade-offs during larval development [32,35]. 

On some occasions, changes occurring at the cellular level may be sufficient to prevent changes at the whole-organism level [59]. In the present study, lobster larvae stage I and II maintain similar whole-organism resting metabolism in the different *p*CO_2_ conditions tested while they undergo intense metabolic reprogramming when exposed to OA conditions. During these stages I and II, thirty-two metabolites change significantly between *p*CO_2_ treatments, mainly involving the pathways of unsaturated fatty acids biosynthesis, amino acid metabolism, and fatty acid metabolism. However, the changes induced by the increase in seawater *p*CO_2_ for the main metabolites (e.g., malate, fumarate, succinate, and fatty acids) are different for larvae stage I and II. Fatty acids and citrate cycle intermediates significantly decrease in stage I lobsters as seawater *p*CO_2_ increases, suggesting an increase in energy demand under OA conditions. The exception is represented by succinate, which increases between 800 and 2000 µatm seawater *p*CO_2_. Previous studies in fish and invertebrates reported that OA can reduce mitochondrial capacity and lead to the inhibition of enzymes of the electron transport system and citrate cycle, such as succinate dehydrogenase (complex II) [60,61], which could lead to the accumulation of succinate, as observed, for instance, in oysters [62]. An accumulation of succinate under stressful conditions could also be relevant, considering other functions of succinate in cellular physiology, namely its role in cell signalling, gene expression and enzyme function modulation [63]. An increase in phospho-L-arginine is also a hallmark of exposure to OA in stage I larvae. This amino acid has an important role in buffering high energy phosphate in invertebrates, acting also as a shuttle mechanism for ATP production when needed [64,65].

In stage II larvae, the same metabolites (fatty acids and citrate cycle intermediates) accumulate under 3000 µatm, with no change in succinate or a clear trend in phospho-L-arginine, but with a trend of increasing lactate concentration, suggesting a transition to anaerobic metabolism. An increase in lactate has also been reported in juveniles of several crab species, suggesting the utilisation of anaerobic metabolism to support acid-base regulation under increased seawater *p*CO_2_ [66,67,68]. However, in subadult females *H. americanus* (50–65 mm CL), elevated *p*CO_2_ induced a depletion of L-lactate, which was considered to lead to a putative reduction in oxygen carrying capacity, as L-lactate is a known molecular modulator of hemocyanin’s oxygen affinity [69]. As larvae from stage I to stage III are osmoconformers, their ionic concentration changes according to the environment [7,70]. Thus, changes in environmental *p*CO_2_ may induce considerable changes in the organism’s extracellular fluid acid-base and osmo-ionic status, likely compensated by costly energetic processes: including, for example, ion homeostasis, organic osmolytes pathway regulation, buffering of intracellular [CO_2_] via carbonic anhydrase activity, and mobilisation of carapace carbonates to buffer via bicarbonates dumping the extracellular space. For example, the Dungeness crab *Cancer magister* (Dana, 1852) held under low pH conditions increased ATP production, with a significant impact on amino acid, sugars, and fatty acid metabolic pathways [71]. Moreover, in the green crab *Carcinus maenas* (Linnaeus, 1758) exposure to hypercapnia for two-weeks led to a reduction in intracellular osmolytes such as amino acids, also suggesting disturbances in intracellular iso-osmotic regulation [72]. The increase in betaine observed in stage I larvae exposed to increasing seawater *p*CO_2_ (especially > 1000 µatm) supports the idea that lobsters face cellular osmotic stress at these conditions, as betaine is an intracellular organic osmolyte commonly found in crustaceans [73,74] and other marine animals [75]. Nevertheless, the downregulation of other metabolic pathways, not investigated here, such as those underpinning mineralisation processes, might have caused energetic trade-offs, resulting in comparable resting metabolic rates across the different *p*CO_2_ conditions tested.

### 3.3. Stage III Larvae: The Metamorphosis as a Turning Point in the Ontogeny

Stage III larvae in this study appear to be tolerant to OA. Increases in seawater *p*CO_2_ do not affect their survival, developmental time, mineral contents, or most of the morphological parameters except for cephalothorax length, as also globally shown by Waller et al. [42]. As an exception, the increase in cephalothorax length under OA conditions might be a positive carry-over effect from the exposure of stages I and II to high *p*CO_2_ levels. In fact, an increase in cephalothorax length in developing lobsters has been hypothesized to play an important role in improving ventilation and gas exchange capacity under high *p*CO_2_ conditions [41]. Under OA conditions, possessing an enlarged cephalothorax may support an increase in ventilation capacity, thus potentially enabling an organism to attain a higher level of energy production. This may, in turn, increase the probability for survival and help stage III larvae to prepare for metamorphosis.

From a metabolic point of view, stage III larvae present transitional metabolomics fingerprinting between that of earlier larvae and post-larval and juvenile stages, and an upward trend in resting metabolic rates: the latter showing to increase with an increase in seawater *p*CO_2_. This suggests that this larval stage is a metabolic turning point in the ontogeny of the American lobster, as the larvae prepare to undergo metamorphosis and they acquire greater osmoregulatory abilities [4,70], both processes being highly energetically demanding. This increase in energy requirements may explain the slight increase in metabolic rates that is observed at stage III under OA conditions. The latest trend was likely highlighted as we applied a regression approach with a seven *p*CO_2_ level gradient. Other, studies testing a smaller number of *p*CO_2_ conditions, and which did not employ an individual-based measurement philosophy, have to date not shown any change in oxygen consumption for stage III lobster larvae exposed to OA [34,42]. An activation of the specific pathway of glycine/serine/threonine metabolism was detected at stage III, with betaine concentrations rising with the increase in seawater *p*CO_2_. This corroborates the suggestion that stage III lobsters exposed to elevated *p*CO_2_ face osmotic (and likely including acid-base) challenges, by compensating via the upregulation of intracellular organic osmolytes, as also described for stage I larvae above.

### 3.4. Passed Metamorphosis: Post-Larval and Juvenile Stages Can Cope with Elevated pCO_2_ but Are More Sensitive Than Larvae

Metamorphosis of stage III larvae results in post-larval stage IV, which is considered as the first manifestation of juvenile traits along the American lobster ontogeny [4]. Survival rates recorded in stage IV lobsters (>30% in all the *p*CO_2_ conditions) in our experiment are higher than those measured in other experiments: 2.6% in the American lobster at 19 °C [42], and 0.5% in the European lobster at 17 °C [34]. Stage V juveniles show an approximate two-fold increase in mortality compared to previous life stages, increasing with elevated *p*CO_2_ (only 10% survival at the highest *p*CO_2_ levels tested), as similarly reported for the European lobster [76]. Finally, the survival rate of stage VI is between 0 and <5% (*p*CO_2_ 600 and 1000 µatm), this value being close to the very low successful settlement rate estimated via modelling for American lobster post-larvae (∼2.5%, [77]).

In parallel, intermoult periods between stage IV–V and V–VI increase under OA conditions in the juvenile stages, as already shown by Menu-Courey et al. [41]. It is known that the time at which settlement behaviour first appears in post-larvae can vary according to environmental conditions [78]. The increase in *p*CO_2_ seems to postpone the settlement of the post-larval stage IV and their transformation into stage V juveniles, leading to a longer planktonic phase which may increase the predation risk [39,79]. The significant effect of elevated *p*CO_2_ conditions (>1200 µatm) on juvenile survival and developmental time supports the theory that juveniles are more sensitive to OA than larvae, as also described by Waller et al. [42].

The formation of the calcified exoskeleton is a costly energetic process [21]. This process is usually one of the first that is down-regulated under elevated *p*CO_2_ conditions in order to reallocate energy to other avenues, resulting in mineralisation changes or carapace length reductions [32,76]. No significant change in carapace cation contents or [Mg^2+^]:[Ca^2+^] is shown under elevated *p*CO_2_ scenarios in our study. We provide no evidence of any change in morphometric traits, with the exception of abdomen length, which slightly changes with increasing *p*CO_2_, shortening for stage IV and extending for stage V. This lack of strong changes in this parameter differs from the general reduction trend that has been reported for lobster juveniles exposed acutely to OA and carbon capture storage (CCS) leakage conditions [40,41,76].

Resting metabolic rates of the American lobster post-larvae and juveniles were not significantly affected by the increase in *p*CO_2_, as previously demonstrated in the same species [41,42], but also for other crustaceans [80,81,82] under OA scenarios. This capacity to face OA conditions, even extreme ones found in coastal areas or potentially caused by CCS leakages, without undergoing metabolic depression may be related to efficient homeostasis and/or acid-base buffering evolved by crustaceans [28,83,84] and underpinned by gene upregulation [36].

In juveniles of the American lobster, the lack of changes in resting metabolic rates, mineralisation compounds, and feeding rate tends to indicate that there is no apparent physiological compensation or trade-offs in response to increasing levels of seawater *p*CO_2_. This finding is further supported by the absence of metabolomics reprogramming at this stage. Interestingly, the capacity for molecular reprogramming (i.e., metabolomic plasticity) may ultimately prevent changes at the whole-organism level [59,85], which seems to be the case for early larvae, as opposed to post-metamorphic lobsters. The lack of metabolomic plasticity to OA and CCS leakage conditions in stage IV and V provide a mechanistic basis for the inability of juvenile lobsters to cope with OA, shown by our study and others before [42,76]. As the increase in *p*CO_2_ levels delays the moult and leads to higher mortality rates in stage V and VI without apparent physiological adjustment, the juveniles of the American lobster appear quite sensitive to elevated seawater *p*CO_2_ conditions mimicking future coastal acidification or CCS leakages.

## 4. Materials and Methods

### 4.1. Specimen Collection

Berried females of the American lobster *Homarus americanus* (H. Milne Edwards, 1837), were captured in late in Spring 2016 off the coast of Shippagan (NB, Canada) in the Baie des Chaleurs (47°46’47″ N 64°42’49″ W) and held at the Coastal Zones Research Institute (CZRI, Shippagan, NB, Canada, now named Valores) in highly aerated 1500 L tanks filled with seawater pumped in front of the CZRI. Newly hatched larvae (stage I) were released after female exposure to a thermal shock and collected early in the morning with big nets. Then, they were transported by car over a 5 h driving distance, in continuously aerated 60-L coolers (Coleman Company Inc., Kingfisher, OK, USA) filled with ambient seawater (18 °C, salinity 34) from the CZRI. Larvae were then transferred in the experimental system at the Department of Fisheries and Oceans facility at the Saint Andrews Biological Station (SABS, Saint Andrews, NB, Canada).

### 4.2. Experimental Design, Setup and Protocol

In order to verify the impacts of the increase in seawater *p*CO_2_/decrease in pH on life-history and physiological traits of early life stages of the American lobster throughout their ontogeny, early life stage individuals were exposed to seven *p*CO_2_ levels corresponding to different pH_T_ (pH on the total scale) from the first larval stage to the stage V juvenile in a larval and then a juvenile setup. These treatments were selected to mimic current *p*CO_2_ condition (400 µatm), predicted values that could be encountered by coastal organisms under ocean acidification scenarios or during upwellings (600, 800, 1000, 1200, and 2000 µatm) and extreme coastal acidification or carbon capture storage leaks (3000 µatm) [13,17,86,87].

In turns, six hundred stage I larvae were transferred by gently siphoning from the cooler to each of the twenty-eight 60 L kreisel tanks (hereafter called “kreisel”) where pelagic larvae were housed during the exposure period (density 10 larvae L^−1^). Lobster larvae were grown from stages I to IV in this system between the 1st and the 18th of July 2016. After metamorphosis (stage IV to stage V), 12 stage IV juveniles from a subset of three kreisels per *p*CO_2_ condition were transferred in the twenty-one 500 L tanks (hereafter called “tanks”) from the juvenile setup. Individuals from the same kreisel were assigned to one specific tank where they grew up from the 18th of July until the 21st of August 2016. Juveniles were gently scooped up using polypropylene cups (Disposable Polypropylene Graduated Medicine Cup, SETON Canada, Markham, ON, Canada) and then transferred to individual floating basket-like pots (Net Pots, Canadian Wholesale Hypotonics, Elie, MN, Canada; 3.5 inches in diameter) allowing water to flow through them. These containers are permitted to individually recognize and track daily survival, development, and growth of juveniles, as well as prevent cannibalism [88].

Each kreisel and tank continuously received sea water at a rate of 1 L min^−1^ (i.e., a renewal rate of 100% h^−1^) from three header tanks where temperature was maintained around 18 °C with heaters and a photoperiod of 16:8. These header tanks were supplied with 20 µm sand-filtered UV sterilized seawater continuously pumped in Passamaquoddy Bay (45°04′60.00″ N-67°04′60.00″ W) in front of SABS, between 10 and 30 m depth, depending on the tide.

Each of the seven *p*CO_2_ treatments had four replicates for the 60 L-kreisel system and three replicates for the 150 L-tank system. The different conditions were gradually reached over the first day of larval transfer to avoid any pH shock. The autonomous feedback system (IKS Aquastar, Karlsbad, Germany) was used to reproduce the different *p*CO_2_ treatments (600, 800, 1000, 1200, 2000, and 3000 µatm) and adjusted the corresponding pH values by bubbling pure CO_2_ to maintain the required conditions independently in each kreisel or tank. The ambient treatment (400 µatm) had no gas regulation and the pH measured was representative of that found in Passamaquoddy Bay. The pH values of the IKS system were adjusted from daily measurements of pH_T_ in the 28 kreisels and 21 tanks using a pH meter (SevenGo pH meter MT, Mettler Toledo AG, Schwerzenbach, Switzerland), coupled to a pH/temperature probe (InLab 413 SG/2m, Mettler Toledo AG), and calibrated with Tris HCl buffer [89]. The temperature was kept constant in the system, at 18 °C, representing the measured water temperature in the Baie des Chaleurs, St Lawrence Gulf (Shippagan, NB, Canada) when larvae were released.

Larvae were fed ad libitum, every day with frozen *Artemia* (Brine Shrimp, Kyorin Co. Ltd., Tokyo, Japan). Stage I received 10 g twice a day at a concentration of approximately 0.3 g larva^−1^ d^−1^. Stage II and III received 5 g twice a day as the larval density decreased in the kreisels (similar concentration food as for stage I). During the day, the food was eaten by the larvae, limiting potential bacterial development and the remaining pieces stuck to the kreisel walls were removed every day with a paper towel. When juveniles were transferred in the individual pots, they were fed ad libitum with small pieces of fish. In turn, thawed cubes of herring (*Clupea harengus* Linneaus 1758) were replaced in the pots every 2 d to keep the food fresh and avoid any bacterial contamination.

As soon as 95% of the individuals reached the following stage, individuals were gently scooped up in the kreisels/tanks and then transferred in vials for resting metabolism determination (see below).

### 4.3. Seawater Parameter Monitoring

Seawater parameters were monitored throughout the experiment. Temperature, dissolved oxygen (% air saturation) and pH_T_ were recorded daily in each of the 28 kreisels and 21 tanks using a dissolved oxygen meter (SevenGo Pro, Mettler Toledo AG) and a pH/temperature meter (SevenGo pH meter MT, Mettler Toledo AG) coupled to a calibrated pH/temperature probe (InLab 413 SG/2m, Mettler Toledo AG) as described above. Seawater samples (500 mL) were randomly taken in one of each kreisel and tank per *p*CO_2_ condition every week. Samples for total alkalinity and (TA) and dissolved inorganic carbon (DIC) were fixed with HgCl_2_ (0.02%) to eliminate microbial activity, stored in 500 mL borosilicate flasks, and maintained in dark and dry conditions until they were shipped to the Bedford Institute of Oceanography (BIO, Dartmouth, NS, Canada) where they were analysed. Salinity was measured at BIO using a conductivity/salinity meter (YSI EC300A, YSI Inc./Xylem Inc., Yellow Springs, OH, USA—calibrated with Ricca conductivity solutions). TA was analysed by open cell potentiometric titration (Metrohm Titrando with an 800 Dosino, Metrohm AG, Herisau, Switzerland). DIC was determined using gas extraction from acidified samples with coulometric quantification of CO_2_ (Dickson et al., 2007).

Partial pressure of CO_2_ (*p*CO_2_)_,_ bicarbonate (HCO_3_^−^) and carbonate (CO_3_^2−^) ion concentrations and the saturation state of calcite (Ω_Ca_) and aragonite (Ω_Ar_) were calculated on daily samples for each kreisel and tank with CO2SYS software [90] using constants from Mehrbach et al. (1973) refitted by Dickson and Millero (1987). The mean ± standard error (SE) results for each *p*CO_2_ treatment and for both larval and juvenile setups are reported in Table S7.

### 4.4. Survival

Survival rate expressed as the percentage of live individuals compared to the number of larvae introduced into the kreisel at the beginning of the experiment (i.e., 600 larvae) was determined every 2 d in each kreisel. Larval survival was calculated as the average of live larvae counted in four subsamples of 500 mL for stage I, and 1 L for stage II and III, rescaled to the volume of the kreisel, and then divided by the total of larvae introduced at the beginning of the experiment [42]. Individuals removed (and not replaced) for metabolic measurements were also taken into account. Sampling every 2 d allowed for larval intermoult-period lengths to be determined. Juvenile condition (i.e., ‘alive’, ‘dead’, or ‘moulted’) was visually checked every day after individuals were transferred in floating basket-like pots. As for larvae, survival rate was expressed as the number of individuals transferred from the larval system (12 per tank), taking into account individuals that were not replaced for metabolic measurements.

### 4.5. Feeding Rate (Only for Juveniles)

Feeding rates (FR) were assessed for the stage IV post-larvae and stage V juveniles, between 5 and 8 d after the moult from the previous stage was completed, on the same individuals used later for MO_2_ measurements. Individuals were not fed for 24 h, and then fed with pre-weighed and paper-blotted blocks of herring *C. harengus*. After 1 h, non-consumed food was removed from the pots and weighed again. Feeding rates (in mg h^−1^) were calculated as:

(1) $$\mathrm{FR}\;=\frac{\Delta W\;}{\Delta t\;}$$

where ΔW (in mg) is the difference between initial and final herring weight, and Δt (in h) is the feeding time.

### 4.6. Resting Metabolic Rates (MO_2_)

Oxygen consumption rates (MO_2_), used as a proxy for resting metabolic rates [91] were measured at each life stage, during the intermoult (7 to 10 d after the moult was completed and the carapace had hardened). For stages I, II, and III, 20 larvae per *p*CO_2_ condition (i.e., 5 per kreisel from that *p*CO_2_ condition) were scooped up in 500 mL beakers. They were then gently transferred with plastic pipettes in glass vials filled with 0.7 µm (GF/F) filtered sea water (Whatman^®^ glass microfiber filters grade GF/F 0.7 µm, GE Healthcare, Chicago, IL, USA) mixed between the kreisels from a same *p*CO_2_ condition. Larvae were incubated individually in 6.3 ± 0.1 mL vials, containing filtered sea water at the same temperature and *p*CO_2_ to which the individual had been previously exposed. In the vial, the larva was placed onto a plastic grid which separated it from a magnetic flea used to mix the water inside. For stages IV and V, six juveniles per *p*CO_2_ condition were not fed for 24 h prior to the measurement of oxygen to minimise the influence of feeding history and digestive activity [92]. Individuals were carefully transferred to 16.9 ± 0.1 mL vials, used as respiration chambers, the day before measuring MO_2_ in order to reduce the stress induced by the new environment. They stayed overnight in their own 150 L tanks, in floating vials sealed with mesh, allowing the water to flow through to avoid hypoxia. In each vial, the juvenile was separated from the magnetic flea by a net which constrained its movement to a 3 cm-length section from the cap.

Determination of oxygen consumption rates was carried out over a 90-min period for stage I to III and over a 60-min period for stage IV and V. Previous tests were run to be sure that the oxygen consumption of each larval and juvenile stage was linear between 100 and 30% air saturation in the vial. Blank incubations, containing only filtered sea water from the kreisels or water from the tank, fleas, and grids used in the vials, also helped to correct fluxes for any microbiological activity and background respiration in seawater. Vials fitted with magnetic flea (speed 350 rpm) were placed in a container for incubation over a magnetic stirrer plate (Mix 15, 2Mag AG, Munich, Germany), to ensure water homogeneity and keep the temperature constant. Incubations were carried out in a temperature-controlled room, with red light to avoid lobsters responding behaviourally to visual stimuli whilst in incubation [93]. Oxygen concentrations were measured at the beginning and the end of the incubation period with a non-invasive fiber-optic system and reactive oxygen spots attached to the inner wall of the chambers (FIBOX 4, PreSens, Regensburg, Germany). The optical fiber was calibrated at 18 °C with 0% and 100% oxygen buffers. Resting metabolic rates called MO_2_ (in µmol O_2_ h^−1^) were calculated from the decline in oxygen concentration per unit time:

(2) $$\mathrm{MO}2\;=\frac{{\Delta O}_{2}\times V\;}{\Delta t\;}\;$$

where ΔO_2_ (in µmol O_2_ L^−1^) is the difference between initial and final O_2_ concentrations; V (in L) is the volume of vial; and Δt (in h) is the incubation time.

### 4.7. Morphological Traits

All larvae and juveniles used for metabolic rates were laid on their right-hand side under a binocular (M80, Leica Microsystems GmbH, Wetzlar, Germany) to be photographed under at magnification ×7.5 with a picture acquisition system (IC80 HD, Leica Microsystems GmbH). Pictures were then analysed with the ImageJ software (Rasband, WS, US National Institutes of Health, Bethesda, MD, USA) in order to determine individuals’ measurements for total body length (BL: tip of rostrum to end of uropods, mm), carapace length (CL: rear of eye socket to rear of carapace, mm), abdomen length (AL: front of first abdominal segment to rear of last abdominal segment, mm), tail length (TL: rear of last abdominal segment to rear of the tail, mm), and chaela length (ChL, mm).

Wet body mass (WBM, g) was measured at a precision of ±1 mg using an electronic scale (ENTRIS 623-19, Sartorius Lab Instruments GmbH KG, Goettingen, Germany) after the excess of water was removed by gently blotting individuals with fine tissue paper (Kimwipes, Kimberly Clark Professional, Roswell, GA, USA). The consecutive tasks of taking a picture, blotting, and weighing took approximately 2 min for each individual after they have been removed from their vial. Entire larvae were then directly flash frozen using liquid nitrogen while the cephalothorax, abdomen, and claw of the juveniles were separated using plastic tools (White Plastic Tweezers, Swiss Precision Instrument Inc., Garden Grove, CA, USA) in order to prevent metal contamination. Samples were stored at −80 °C to preserve them for carapace mineralisation and metabolomics analyses (see below).

### 4.8. Carapace Mineralisation

Chemical analyses on the carapace mineralisation status of the lobster larvae and juveniles, as an important proxy for changes in the composition of this organ in crustaceans under low pH seawater conditions, were performed at the Laboratoire de Chimie Marine et Spectrométrie de Masse at the University of Quebec in Rimouski (Rimouski, QC, Canada). Previously frozen entire larvae and juvenile’s cephalothorax carapace were removed using non-metal tools (White Plastic Tweezers, Swiss Precision Instrument Inc., Garden Grove, CA, USA) and freeze dried for 12 h to remove any residual moisture (Freezone Freeze Dry Systems, Labcono, Kansas City, MO, USA). Samples were then weighed on a high precision microbalance (MX5 Analytical Micro-balance, Mettler Toledo). Hereafter, they were digested in a mixture of pure nitric acid and peroxide hydrogen (375 µL:125 µL) (TraceSelect grade, Sigma Aldrich, St. Louis, MO, USA) at room ambient temperature for 24 h and after short periods of sonication and warming. Samples diluted in ultrapure water were analysed by inductively coupled plasma (ICP) interfaced to a quadruple mass spectrometer (MS-ICP-MS, Agilent 7500c with micro flow nebulizer, Agilent Technologies, Santa Clara, CA, USA) equipped with an autosampler (ASX 520, Teledyne CETAC, Omaha, NB, USA). Element signals were acquired for 200 msec per mass, and three acquisitions were realized. Element quantification ([Sr^+^], [Ca^2+^], [Mg^2+^], [Na^+^], and [K^+^]) was performed in normal mode with a ten points external calibration using multi-element reference material (Multi-Element 5, Sigma Aldrich) with a concentration range between 0.10 and 50 for [Sr^+^] and 1.00 and 500 ng mL^−1^ for other elements. Performances of the method and instrument stability were assessed by the analysis of a quality control (QC) solution of known metal concentration repeatedly during the course of analysis and a procedural blank. Control of the system, acquisition, and data processing were carried out with the Agilent ChemStation software (Agilent Technologies). [Mg^2+^]:[Ca^2+^] were calculated, as these two ions, in the form of carbonates, are essential components of the carapace.

### 4.9. Statistical Analyses for Life-History and Physiological Traits

All statistical analyses were performed using the R software, version 2.15.0 (R Core Team 2013, R foundation for Statistical computing, Vienna, Austria). Cumulated survival was analysed by a binomial model weighted by the initial effective of larvae or juveniles. For all the other data analysed, normality and homoscedasticity of the data and residuals were checked using Shapiro’s test and Bartlett’s test, respectively. Data were log transformed to respect the assumptions for the mineralisation traits. Linear mixed effect models [94] were built for each variable with “*p*CO_2_” and “stage” as fixed factors. The wet body mass (for MO_2_, feeding rate, and mineralisation traits) or the total length (morphometric traits except ratios) of each individual, and the mean temperature measured during the experiment (for MO_2_), were added as covariates. Spatial pseudoreplication was taken into account by considering “kreisel” or “tank” as random factors for all variables tested. The significance of the random term was first tested with the function “rand” of the package lmerTest [95], and the random term was removed as it was non-significant for all parameters tested. After removing the random term, linear, log, and polynomial models were compared with Akaike information criteria (AIC). The best model was determined as the one with the lower AIC: however, ΔAIC were calculated, and for models with ΔAIC < 2, thus considerably comparable, the best model has been chosen as the most parsimonious one, i.e., the simplest order equation type, e.g., linear over log over polynomial 2 degrees over polynomial 3 degrees. In case significant interaction between *p*CO_2_ and stage was found, the best model was used to predict the responses and assess the significance of the trait to *p*CO_2_ for each stage, fixing the value of the covariate as the mean value for each stage.

All results in the tables are given as mean ± standard error (SE) and the graphs represent the predicted response to *p*CO_2_ flanked by the confidence interval for each developmental stage, except for the graph presenting cumulative survival (mean ± SE).

### 4.10. Metabolomic Fingerprints

Metabolite quantification: The extraction of key targeted metabolites was carried out on previously frozen entire larvae and juvenile’s abdomen (same individuals used for respiration measurements, *n* = 20 and 6, respectively), following the method described by Lu et al. [96] and the adapted protocol detailed in [97]. Briefly, a fast “cold quenching salt-eliminating” extraction was first prepared, using an ammonium carbonate extraction solution. Centrifugal PP tubes (1.5 mL) containing the samples were kept in liquid nitrogen, while 250 µL of the extraction solution were added in the tube. Each sample was crushed with a potter pestle (blue pre-sterilized, Axygen, Tewksbury, MA, USA), sonicated for 3 s (Sonication bath, model Symphony, VWR, West Chester, PA, USA) and centrifuged at 11,000 rpm for 3 min at 4 °C (centrifuge 5430R, Eppendorf, Hamburg, Germany). After transfer in a 250 µL amber HPLC vial with insert (Wheaton, NJ, USA), 225 µL of the supernatant were injected in a liquid chromatography system (Accela, Thermo Electron Corporation, San Jose, CA, USA) equipped with a 150 mm × 2 mm Luna C5 guard column for Phenomenex (Torrance, CA, USA). The identification of metabolites previously separated was then achieved on an Orbitrap LTQ Discovery high-resolution mass spectrometer (HRMS) (Thermo Electron Corporation). HRMS data were then analysed on Xcalibur 2.0 software (Thermo Electron Corporation). For each targeted metabolite, a calibration curve was created using amino-acid (from Phenomenex), free fatty-acid (from hydrolyzed FAME 37 standard, Sigma-Aldrich), and other metabolites standards (Sigma-Aldrich). Metabolite concentrations for all the samples were then assessed from the area of the working standard solution by extraction integration.

Data pre-processing: Raw data matrices were blank subtracted (a mean blank value was calculated per metabolite) and normalized by wet weight, with final concentration values expressed as ng metabolite mg^−1^ wet weight. Matrices were then imported into the freely available web tool MetImp v1.2 (https://metabolomics.cc.hawaii.edu/software/MetImp/, accessed in April 2021) for missing data imputation. Following the protocol established for targeted LC-MS [98], the QRILC algorithm (Quantile Regression Imputation of Left-Censored Data) with 50% group wise missing filtering was used to input missing values (seed 1234). Afterwards, data were imported to Metaboanalyst 4.0 (http://www.metaboanalyst.ca/, accessed in April 2021), log-transformed to reduce heteroscedasticity and pareto-scaled to adjust for differences in fold-changes between metabolites.

Data analysis: Multivariate and univariate analyses were performed to identify metabolites that discriminate between (i) developmental stages and (ii) different *p*CO_2_ concentrations within each stage. Unsupervised principal components analysis (PCA) was applied to unravel data structure, and was followed by a supervised method, namely partial-least-squares discriminant analysis (PLS-DA) in order to identify which metabolites are useful to predict group membership, following the rationale described in Worley and Powers [99,100]. Metabolites with discriminative power were ranked based on VIP values > 1 (Variable Importance in Projection score) and PLS-DA models were validated based on the “prediction accuracy during training” test statistic with 1000 permutations (*p* < 0.05 for significance). Heat maps with hierarchical clustering of metabolites were constructed based on the following metrics (i) distance measure: Pearson correlation (similarity of expression profiles), (ii) clustering algorithm: complete linkage (forms compact clusters), and (iii) feature autoscale. Hierarchical clustering of samples was carried out based on the following metrics: (i) distance measure: Euclidean distance (sensitive to magnitude differences), and (ii) clustering algorithm: Ward (minimizes within-cluster variance). Differences in metabolites between groups were further evaluated using one-way analysis of variance (ANOVA) with a False Discovery Rate (FDR) cut-off set at 0.05 for significance. Tukey’s post hocs were applied to check which groups differed. Significant metabolites unravelled by ANOVA were then used for pathway analysis to identify the most relevant pathways that are involved in developmental processes and responses to seawater *p*CO_2_. As there are no libraries available for lobster, the pathway library used was from the model species *D. rerio* (zebrafish), and analysis was based on homology. Pathway analysis was carried out based on two features, (i) functional enrichment, which was assessed using hypergeometric tests for over-representation analysis (*p* < 0.05 for significance), and (ii) pathway topology analysis, which was implemented using the relative betweenness centrality. Pathway topology takes into account the role of the metabolite, position and direction of the interaction, measuring the centrality of a given metabolite in the metabolic network. This gives information about the relative importance or role of the metabolite in the organization of the metabolic network. Indeed, central metabolites are assumed to have primary functions in metabolism when compared to peripheral metabolites, as they control other metabolic reactions and, thus, network flow, having greater downstream consequences in cellular functioning—see Xia and Wishart [101] for further details. Pathway impact was considered relevant if >0.1.

## 5. Conclusions

Our study further confirms the low sensitivity of the larval stages of the American lobster to OA and CCS leakage conditions (up to *p*CO_2_ 3000 µatm/pH 7.2), conversely to the historically accepted idea that early larvae would be the most sensitive life stages to elevated seawater *p*CO_2_ [48,102]. Stage IV post-larvae and stage V juveniles possess the narrowest tolerance window to OA. *Homarus americanus* stage IV post-larvae in particular, the transitional bentho-pelagic stage, appear to be one of the main bottlenecks along crustacean development (as observed in decapod crustaceans: e.g., [34,35]). This has likely profound ecological and population dynamics implications for the American lobster, as observed for other crustaceans: e.g., the estuarine crab *Chasmagnathus granulata* (Dana, 1851) [103] and the barnacle *Semibalanus balanoides* (Linnaeus, 1767) [104].

Our study provides evidence that the ability to activate cellular compensatory mechanisms can minimize the negative impacts of high *p*CO_2_ conditions on life history and whole-organism physiological responses, as observed in our study for larval stages. Interestingly, a similar pattern was observed at the gene expression level by Niemisto et al. [36]. In more detail, we show that larvae OA buffering capacity in the American lobster is connected to their ability to regulate energy metabolic pathways related to fatty acid metabolism, amino acid metabolism, and citrate cycle metabolism.

Future experiments should continue the efforts to investigate life history, whole organism physiology and cell responses over discrete pH/*p*CO_2_ gradients, as we achieved in this study. In addition, future studies should attempt to further integrate multi omics approaches, to deepen our mechanistic understanding of marine species responses to global changes, whilst extending the exposure to OA and CCS conditions over further life stages, and even over multiple generations. Indeed, it is still to be determined if the impacts observed for early life stages will translate into impacts at the adult stages, and if there will be longer-term effects on population fecundity due to maternal carry-over exposures, as well as across multiple-generations of exposures. Finally, there is an urgent need to acquire an in-depth understanding of marine invertebrates’ stage-specific physiological sensitivity to future global change drivers at different scales of the biological hierarchy, renewing Bartholomew’s lesson [105], particularly for species characterised by complex life-cycles.

## Figures and Tables

**Table 1:** **Models and statistics.** Summary of best model fitted testing the effect of *p*CO_2_, stage and their interaction on the American lobster *Homarus americanus* life history and physiological traits. Numbers in bold indicate significant *p*-values. Lt: total length and wbm: wet body mass. Survival between stages is calculated as the percentage of survival at each stage, assuming 100% corresponds to the number of larvae at the beginning of each larval stage. Developmental time was only determined for stages IV and V.

	Best Model Fitted												
	*p*CO_2_			Stage			*p*CO_2_: Stage			Covariate			
	df	F	*p*	df	F	*p*	df	F	*p*	df	df	F	*p*
Survival cumulated	1	31.2	**<0.001**							no			
Survival between stages	1	29.87	**<0.001**	4	2129.87	**<0.001**	4	666.51	**<0.001**	no			
Developmental time	1	11.572	**<0.001**	1	15.884	**<0.001**	1	0.908	0.342	no			
Morphometrics													
Total length (Lt)	1	13.981	**<0.001**	4	582.854	**<0.001**	4	0.327	0.860	no			
ceph/Lt	1	0.317	0.574	4	94.258	**<0.001**	4	1.947	0.102	no			
abd/Lt	1	2.291	0.131	4	23.342	**<0.001**	4	1.673	0.155	no			
ceph/abd	1	1.436	0.232	4	65.443	**<0.001**	4	1.434	0.222	no			
Rostrum	1	3.733	0.054	4	14.608	**<0.001**	4	1.467	0.211	Lt	1	356.727	<0.001
Cephalothorax	1	0.330	0.568	4	40.443	**<0.001**	4	2.837	**0.024**	Lt	1	5075.599	<0.001
Abdomen	1	0.847	0.358	4	21.266	**<0.001**	4	3.538	**0.007**	Lt	1	2885.833	<0.001
Tail	1	0.007	0.932	4	24.358	**<0.001**	4	4.526	**0.001**	Lt	1	1095.440	<0.001
Claw	1	1.166	0.281	4	120.891	**<0.001**	4	0.793	0.530	Lt	1	4108.430	<0.001
Mineralisation													
log(Ca^2+^)	1	0.766	0.384	4	5.955	**<0.001**	4	1.478	0.215	wbm	1	55.695	<0.001
log(Mg^2+^)	1	4.357	0.040	4	2.962	**0.024**	4	2.748	**0.033**	wbm	1	0.334	0.565
log(Na^+^)	1	5.137	0.026	4	2.111	0.086	4	2.720	**0.034**	wbm	1	102.544	<0.001
log(K^+^)	1	4.665	0.033	4	3.970	**0.005**	4	1.753	0.145	wbm	1	95.232	<0.001
log(Sr^+^)	1	0.541	0.464	4	5.849	**<0.001**	4	1.257	0.293	wbm	1	49.116	<0.001
[Mg^2+^]:[Ca^2+^]	1	5.049	0.008	4	19.040	**<0.001**	4	2.316	**0.026**	wbm	1	125.105	<0.001
Feeding rate	1	0.169	0.683	4	1.620	0.209	4	1.143	0.290	wbm	1	5.377	0.024
Resting metabolic rate (MO_2_)	1	1.608	0.202	4	4.440	**0.002**	4	3.210	**0.001**	wbm	1	1196.088	<0.001

## Data Availability

All data from this article will be available on the Open Access Repository PANGAEA, under OA-ICC data compilation, DOI TO COME.

## Supplementary Materials

The following are available online at https://www.mdpi.com/article/10.3390/metabo11090584/s1, Figure S1: Metabolomic fingerprinting stages III to IV. Metabolome differences between *p*CO_2_ groups in lobster larval stages III, IV and V (a) PCA 2D score plot with 95% confidence intervals for stage III, (b) PCA 2D score plot with 95% confidence intervals for stage IV, (c) PCA 2D score plot with 95% confidence intervals for stage V, (d) PLS-DA scores plot with 95% confidence intervals for stage V. Model validation was calculated by the prediction accuracy during training test statistic with 1000 permutations, *p* = 0.016, (e) PLS-DA Variable Importance in Projection scores (VIP > 1 for significance). None of the metabolites were significant in the univariate ANOVA (FDR 0.05). *p*CO_2_ groups: 1—400 µatm, 2—600 µatm, 3—800 µatm, 4—1000 µatm, 5—1200 µatm, 6—2000 µatm, 7—3000 µatm. Groups 6 and 7 are lacking from stage V analysis due to mortality levels., Table S1: Stage survival and morphological measurements for each *p*CO_2_ condition of exposure at each stage of development, Table S2: Mineral contents in the cephalothorax for each *p*CO_2_ condition of exposure at each stage of development, Table S3: Resting metabolic rates (individual and mass normalized) for each *p*CO_2_ condition of exposure at each stage of development. n = 20 for larval stages I, II, III and n = 6 for postlarvae (stage IV) and juveniles (stage V), Table S4: Models testing the responses of life-history and physiological traits to *p*CO_2_ and stage (+covariate when needed). AIC, R² and adjusted R² are given for each equation. Bold R² shows the best fitted model based on AIC selection for each trait, following the rule of parsimony when AIC differ for less than a value of 2: i.e., when AIC are comparable the simpler model was consider as the best-fit model, Table S5 ANOVA and post-hoc Tukey HSD results assessing the effects of ocean acidification (*p*CO_2_ concentration groups: 1—400 µatm; 2—600 µatm; 3—800 µatm; 4—1000 µatm; 5—1200 µatm; 6—2000 µatm; 7—3000 µatm) in stage I larvae. Only significant metabolites are included in the table, Table S6. ANOVA and Tukey HSD post-hoc results assessing the effects of ocean acidification (*p*CO_2_ concentration groups: 1—400 µatm; 2—600 µatm; 3—800 µatm; 4—1000 µatm; 5—1200 µatm; 6—2000 µatm; 7—3000 µatm) in stage II larvae. Only significant metabolites are included in the table, Table S7: Physico-chemical parameters (mean ± SE) of the different *p*CO_2_ treatments for larval setup (nkreisels = 4 per *p*CO_2_ condition) and juvenile setup (ntanks = 3 per *p*CO_2_ condition). Seawater temperature, salinity, oxygen content, pHT (pH on the total scale) and total alkalinity (At) were measured whereas the other parameters of the carbonate system were calculated with CO_2_sys software (Lewis & Wallace, 1998). *p*CO_2_: CO_2_ partial pressure; DIC: dissolved inorganic carbon; HCO^3−^: bicarbonates concentration; CO_3_^2−^: carbonates concentration; Ω Ca: saturation state of seawater with respect to calcite and Ω Ar: saturation state of seawater with respect to aragonite.
