# Supplementary material for: Tolerant Larvae and Sensitive Juveniles: Integrating Metabolomics and Whole-Organism Responses to Define Life-Stage Specific Sensitivity to Ocean Acidification in the American Lobster
Source: Metabolites. 2021 Aug 30;11(9):584. doi: 10.3390/metabo11090584 (PMC8467062; doi:10.3390/metabo11090584)

Supplementary Material

Table S1: Stage survival and morphological measurements for each pCO_2 condition of exposure at each stage of development.

stage	pCO_2		Survival (%)		Rostrum (mm)		Cephalothorax (mm)		Abdomen (mm)		Tail (mm)		Claw (mm)		Total length (mm)		Abdomen / Total length		Cephalothorax / Total length		Cephalothorax /Abdomen	
	μatm				Mean	SE	Mean	SE	Mean	SE	Mean	SE	Mean	SE	Mean	SE	Mean	SE	Mean	SE	Mean	SE
I	400	20	51	13	1.61	0.04	1.79	0.03	2.68	0.05	1.33	0.05	0.81	0.04	4.47	0.07	0.60	0.01	0.40	0.01	0.67	0.02
I	600	18	54	7	1.54	0.04	1.88	0.06	2.69	0.08	1.33	0.04	0.89	0.05	4.57	0.12	0.59	0.01	0.41	0.01	0.71	0.02
I	800	20	59	7	1.57	0.03	1.90	0.05	2.80	0.06	1.40	0.03	0.75	0.03	4.70	0.06	0.60	0.01	0.40	0.01	0.69	0.03
I	1000	20	47	13	1.46	0.07	2.08	0.10	2.83	0.11	1.31	0.05	0.83	0.05	4.90	0.20	0.58	0.01	0.42	0.01	0.74	0.02
I	1200	18	67	5	1.56	0.04	2.08	0.09	2.91	0.12	1.38	0.05	0.94	0.06	4.99	0.20	0.58	0.01	0.42	0.01	0.72	0.03
I	2000	15	53	8	1.59	0.05	1.92	0.07	2.79	0.10	1.35	0.05	0.77	0.05	4.71	0.14	0.59	0.01	0.41	0.01	0.70	0.03
I	3000	20	78	10	1.48	0.06	1.90	0.08	2.79	0.08	1.34	0.05	0.83	0.03	4.68	0.14	0.60	0.01	0.40	0.01	0.68	0.02
II	400	15	100	0	1.65	0.07	2.30	0.08	3.28	0.13	1.37	0.05	0.94	0.05	5.58	0.20	0.59	0.01	0.41	0.01	0.71	0.02
II	600	17	97	3	1.55	0.05	2.57	0.07	3.32	0.07	1.33	0.06	1.04	0.04	5.90	0.10	0.56	0.01	0.44	0.01	0.78	0.03
II	800	20	76	17	1.51	0.07	2.68	0.05	3.49	0.07	1.39	0.05	1.08	0.04	6.16	0.09	0.57	0.01	0.43	0.01	0.77	0.02
II	1000	14	90	9	1.54	0.09	2.67	0.06	3.25	0.16	1.45	0.07	1.07	0.06	5.92	0.15	0.54	0.02	0.46	0.02	0.88	0.10
II	1200	20	81	10	1.73	0.05	2.39	0.04	3.41	0.07	1.30	0.08	1.07	0.03	5.81	0.09	0.59	0.01	0.41	0.01	0.70	0.02
II	2000	15	96	4	1.60	0.08	2.56	0.06	3.56	0.07	1.37	0.06	1.05	0.04	6.12	0.10	0.58	0.01	0.42	0.01	0.72	0.02
II	3000	15	63	8	1.58	0.06	2.39	0.06	3.30	0.07	1.33	0.05	1.02	0.03	5.69	0.10	0.58	0.01	0.42	0.01	0.73	0.02
III	400	15	61	10	1.97	0.07	2.92	0.05	3.76	0.07	1.63	0.08	1.48	0.06	6.68	0.09	0.56	0.01	0.44	0.01	0.78	0.02
III	600	15	87	10	1.85	0.12	2.78	0.14	3.61	0.15	1.50	0.11	1.41	0.08	6.39	0.28	0.57	0.01	0.43	0.01	0.77	0.02
III	800	20	76	9	1.90	0.10	2.97	0.05	3.85	0.08	1.73	0.06	1.52	0.03	6.81	0.08	0.56	0.01	0.44	0.01	0.78	0.03
III	1000	20	59	9	1.86	0.05	3.01	0.04	3.74	0.08	1.69	0.05	1.48	0.04	6.75	0.09	0.55	0.01	0.45	0.01	0.81	0.02
III	1200	20	86	7	1.97	0.07	2.93	0.05	3.60	0.06	1.68	0.03	1.50	0.04	6.54	0.09	0.55	0.00	0.45	0.00	0.82	0.01
III	2000	14	78	9	2.01	0.09	3.08	0.04	3.81	0.07	1.68	0.03	1.56	0.05	6.88	0.09	0.55	0.00	0.45	0.00	0.81	0.01
III	3000	15	71	10	1.69	0.10	3.15	0.04	3.83	0.05	1.67	0.05	1.47	0.05	6.97	0.08	0.55	0.00	0.45	0.00	0.82	0.01
IV	400	6	23	3	1.94	0.13	3.79	0.08	4.15	0.09	1.76	0.32	3.21	0.19	7.94	0.09	0.52	0.01	0.48	0.01	0.92	0.03
IV	600	5	33	9	1.95	0.08	3.71	0.08	4.00	0.21	1.84	0.04	3.18	0.17	7.72	0.29	0.52	0.01	0.48	0.01	0.93	0.03
IV	800	6	37	9	2.17	0.08	3.78	0.04	4.43	0.11	1.83	0.13	3.29	0.06	8.21	0.15	0.54	0.00	0.46	0.00	0.86	0.02
IV	1000	6	30	12	2.02	0.10	3.64	0.07	3.76	0.06	2.11	0.09	3.00	0.06	7.40	0.08	0.51	0.01	0.49	0.01	0.97	0.03
IV	1200	8	30	4	2.04	0.11	3.65	0.08	3.86	0.12	2.07	0.07	3.09	0.09	7.51	0.18	0.51	0.01	0.49	0.01	0.95	0.02
IV	2000	5	10	6	1.96	0.05	3.64	0.08	3.71	0.13	1.92	0.08	2.82	0.33	7.36	0.19	0.50	0.01	0.50	0.01	0.98	0.03
IV	3000	6	6	6	1.81	0.13	3.73	0.05	4.01	0.10	2.24	0.08	2.99	0.10	7.74	0.11	0.52	0.01	0.48	0.01	0.93	0.03
V	400	5	0	0	2.11	0.06	4.53	0.07	4.63	0.12	2.74	0.09	4.59	0.20	9.16	0.14	0.51	0.01	0.49	0.01	0.98	0.03
V	600	6	7	7	2.22	0.11	4.51	0.12	4.71	0.18	2.54	0.11	4.43	0.21	9.22	0.30	0.51	0.00	0.49	0.00	0.96	0.01
V	800	6	7	7	1.90	0.10	4.74	0.12	4.91	0.17	2.67	0.10	4.50	0.16	9.65	0.27	0.51	0.00	0.49	0.00	0.97	0.02
V	1000	6	7	7	2.23	0.07	4.63	0.18	4.81	0.17	2.57	0.15	4.80	0.24	9.44	0.31	0.51	0.01	0.49	0.01	0.96	0.03
V	1200	8	0	0	2.10	0.11	4.49	0.11	4.90	0.15	2.44	0.12	3.86	0.29	9.38	0.25	0.52	0.00	0.48	0.00	0.92	0.01

Table S2: Mineral contents in the cephalothorax for each pCO_2 condition of exposure at each stage of development.

Stage	pCO_2	n	Ca^{2+} (mg g^{-1} DW)			Mg^{2+} (mg g^{-1} DW)			Na^{+} (mg g^{-1} DW)			K^{+} (mg g^{-1} DW)			Sr^{+} (mg g^{-1} DW)			$[Mg^{2+}]:[Ca^{2+}]$			$[Ca^{2+}]:[Mg^{2+}]$		
			mean		SE	mean		SE	mean		SE	mean		SE	mean		SE	mean		SE	mean		SE
I	400	3	78.35	±	22.69	40.85	±	14.69	153.50	±	14.51	33.22	±	8.26	1.13	±	0.27	0.50	±	0.03	2.04	±	0.13
I	600	3	48.07	±	22.72	13.40	±	6.52	62.80	±	30.13	26.84	±	13.03	0.71	±	0.39	0.23	±	0.06	5.11	±	1.57
I	800	4	59.86	±	11.42	16.51	±	3.56	80.52	±	20.29	32.30	±	6.24	0.75	±	0.15	0.27	±	0.01	3.70	±	0.18
I	1000	4	103.28	±	25.96	23.61	±	3.14	112.84	±	17.54	38.88	±	3.50	1.70	±	0.40	0.25	±	0.03	4.25	±	0.55
I	1200	4	67.35	±	18.77	20.55	±	4.27	133.40	±	13.28	44.75	±	7.73	0.96	±	0.25	0.37	±	0.10	3.17	±	0.63
I	2000	3	79.73	±	31.56	15.11	±	4.59	72.66	±	20.68	21.16	±	4.48	1.26	±	0.52	0.23	±	0.05	4.76	±	0.87
I	3000	4	38.57	±	8.25	12.75	±	2.35	74.83	±	17.39	24.10	±	5.37	0.55	±	0.09	0.35	±	0.06	3.12	±	0.61
II	400	3	121.07	±	40.07	25.13	±	7.17	106.96	±	29.20	37.31	±	9.11	2.05	±	0.67	0.22	±	0.01	4.68	±	0.26
II	600	3	99.05	±	10.17	19.84	±	2.93	95.48	±	20.79	29.41	±	3.04	1.70	±	0.20	0.20	±	0.01	5.07	±	0.26
II	800	4	131.37	±	59.47	30.38	±	11.63	153.49	±	39.28	59.36	±	21.01	2.38	±	1.10	0.47	±	0.25	3.72	±	0.97
II	1000	3	98.12	±	35.02	23.78	±	4.93	112.68	±	2.14	40.76	±	3.23	1.65	±	0.68	0.29	±	0.06	3.82	±	0.74
II	1200	4	83.44	±	17.79	18.50	±	3.30	80.37	±	11.67	33.13	±	6.63	1.53	±	0.32	0.23	±	0.02	4.45	±	0.43
II	2000	3	62.82	±	5.93	12.18	±	1.41	60.84	±	7.99	20.30	±	1.05	1.09	±	0.13	0.19	±	0.01	5.18	±	0.15
II	3000	3	63.85	±	19.90	13.80	±	3.43	55.11	±	14.15	24.73	±	7.59	1.11	±	0.35	0.23	±	0.02	4.50	±	0.34
III	400	3	83.87	±	18.17	13.87	±	4.04	63.58	±	13.64	22.45	±	9.19	1.42	±	0.32	0.16	±	0.01	6.35	±	0.51
III	600	3	101.58	±	20.43	16.84	±	3.16	76.09	±	17.80	29.00	±	5.04	1.77	±	0.35	0.17	±	0.00	6.01	±	0.16
III	800	4	60.31	±	10.71	10.76	±	2.28	39.26	±	6.89	20.60	±	4.82	1.11	±	0.21	0.17	±	0.01	5.78	±	0.33
III	1000	4	112.36	±	49.08	19.53	±	8.43	82.37	±	31.14	33.03	±	16.55	1.98	±	0.86	0.17	±	0.01	5.85	±	0.36
III	1200	4	142.75	±	83.78	26.85	±	16.08	106.17	±	62.43	53.67	±	32.89	2.63	±	1.56	0.19	±	0.01	5.33	±	0.24
III	2000	3	60.05	±	2.65	9.90	±	0.55	42.68	±	4.69	18.21	±	1.39	1.05	±	0.03	0.17	±	0.01	6.10	±	0.40
III	3000	3	110.01	±	28.80	19.06	±	6.26	79.32	±	26.15	35.07	±	12.96	1.94	±	0.53	0.17	±	0.01	5.99	±	0.52
IV	400	3	197.57	±	82.45	18.08	±	5.66	40.62	±	6.25	14.10	±	3.40	3.12	±	1.28	0.11	±	0.02	10.03	±	1.84
IV	600	3	565.94	±	88.02	48.08	±	33.17	70.85	±	44.73	18.98	±	12.03	8.90	±	6.06	0.08	±	0.00	11.81	±	0.30
IV	800	3	291.77	±	79.99	24.79	±	6.88	47.01	±	11.88	14.11	±	3.56	4.66	±	1.35	0.08	±	0.00	11.81	±	0.42
IV	1000	3	259.40	±	94.96	22.34	±	7.88	46.10	±	11.28	10.16	±	2.80	4.01	±	1.47	0.09	±	0.00	11.52	±	0.16
IV	1200	3	240.06	±	94.81	20.19	±	7.35	33.67	±	11.00	9.26	±	3.75	3.82	±	1.53	0.09	±	0.00	11.57	±	0.49
IV	2000	2	145.78	±	30.68	12.45	±	1.78	24.21	±	0.08	5.79	±	0.38	2.28	±	0.49	0.09	±	0.01	11.60	±	0.81
IV	3000	3	306.36	±	59.27	29.40	±	6.26	60.34	±	8.56	13.90	±	2.09	4.80	±	0.92	0.10	±	0.00	10.49	±	0.36
V	400	3	165.41	±	49.98	17.59	±	6.08	21.17	±	4.02	7.55	±	3.25	2.48	±	0.73	0.10	±	0.00	9.65	±	0.44
V	600	3	343.69	±	68.00	33.16	±	8.20	36.14	±	12.60	14.73	±	5.09	5.26	±	1.04	0.09	±	0.01	10.73	±	0.75
V	800	3	226.97	±	44.46	24.56	±	5.51	34.09	±	9.14	12.61	±	3.12	3.45	±	0.70	0.11	±	0.00	9.37	±	0.31
V	1000	3	227.39	±	22.18	21.69	±	2.13	22.26	±	3.93	9.84	±	1.57	3.37	±	0.34	0.10	±	0.01	10.51	±	0.58
V	1200	4	283.52	±	47.16	25.24	±	3.50	33.66	±	2.44	12.98	±	1.77	4.30	±	0.73	0.09	±	0.01	11.12	±	0.75

Table S3: Resting metabolic rates (individual and mass normalized) for each pCO_2 condition of exposure at each stage of development. n = 20 for larval stages I, II, III and n = 6 for postlarvae (stage IV) and juveniles (stage V).

		Resting Metabolic Rate (µmol O_2 h^{-1})		Resting Metabolic Rate (Mass Normalized) (µmol O_2 g^{-1}WBM h^{-1})	
**Stage**	pCO_2	**Mean**	**SE**	**Mean**	**SE**
I	400	0.13	± 0.01	17.81	± 1.32
I	600	0.16	± 0.01	21.90	± 0.98
I	800	0.15	± 0.01	19.90	± 1.59
I	1000	0.15	± 0.01	16.93	± 1.41
I	1200	0.15	± 0.01	19.90	± 1.44
I	2000	0.14	± 0.01	19.64	± 1.33
I	3000	0.13	± 0.01	18.00	± 1.99
II	400	0.19	± 0.02	17.08	± 2.11
II	600	0.16	± 0.01	15.50	± 0.89
II	800	0.17	± 0.02	15.95	± 1.39
II	1000	0.17	± 0.02	15.41	± 1.96
II	1200	0.18	± 0.01	17.94	± 1.11
II	2000	0.24	± 0.10	16.50	± 3.43
II	3000	0.20	± 0.02	18.96	± 1.66
III	400	0.17	± 0.02	11.22	± 1.30
III	600	0.18	± 0.02	11.09	± 1.22
III	800	0.32	± 0.02	18.29	± 1.01
III	1000	0.16	± 0.02	10.70	± 1.69
III	1200	0.28	± 0.02	16.49	± 1.02
III	2000	0.32	± 0.03	18.04	± 1.06
III	3000	0.31	± 0.02	18.49	± 1.22
IV	400	0.21	± 0.02	6.40	± 0.67
IV	600	0.62	± 0.11	18.49	± 1.87
IV	800	0.23	± 0.04	6.90	± 1.23
IV	1000	0.35	± 0.05	12.87	± 1.56
IV	1200	0.56	± 0.10	18.48	± 3.05
IV	2000	0.46	± 0.03	14.87	± 1.45
IV	3000	0.28	± 0.03	8.96	± 1.06
V	400	0.63	± 0.14	10.94	± 2.14
V	600	0.68	± 0.06	12.03	± 0.93
V	800	0.88	± 0.14	14.24	± 2.19
V	1000	0.80	± 0.09	12.80	± 1.54
V	1200	0.73	± 0.09	13.17	± 1.32

Table S4: Models testing the responses of life-history and physiological traits to pCO_2 and stage (+ covariate when needed). AIC, R^2 and adjusted R^2 are given for each equation. Bold R^2 shows the best fitted model based on AIC selection for each trait, following the rule of parsimony when AIC differ for less than a value of 2: i.e. when AIC are comparable the simpler model was consider as the best-fit model.

	**Linear lm(days~covariate + pCO2*stage**		**Logarithmic lm(days~ covariate + log(pCO2)*stage**		**Polynomial (2nd degree) lm(days~ covariate + poly(pCO2,2)*stage**		**Polynomial (3rd degree) lm(days~ covariate + poly(pCO2,3)*stage**	
	AIC	R^2 (R^2 adjusted)	AIC	R^2 (R^2 adjusted)	AIC	R^2 (R^2 adjusted)	AIC	R^2 (R^2 adjusted)
Developmental time	1011.82	**0.126 (0.113)**	1016.10	0.108 (0.094)	1009.99	0.152 (0.130)	1011.52	0.162 (0.131)
Morphometrics								
Total length (Lt)	995.84	**0.847 (0.843)**	994.95	0.847 (0.844)	995.18	0.850 (0.845)	1000.00	0.852 (0.845)
ceph/Lt	-1975.61	**0.475 (0.464)**	-1975.11	0.475 (0.464)	-1972.90	0.484 (0.467)	-1974.07	0.497 (0.474)
abd/Lt	-1909.59	0.194 (0.177)	-1911.81	**0.198 (0.181)**	-1908.69	0.211 (0.185)	-1901.40	0.216 (0.180)
ceph/abd	-712.68	**0.384 (0.371)**	-712.08	0.383 (0.370)	-707.45	0.390 (0.370)	-705.46	0.401 (0.374)
Rostrum	15.47	**0.501 (0.489)**	17.73	0.498 (0.487)	16.70	0.511 (0.494)	17.22	0.522 (0.498)
Cephalothorax	-95.18	**0.925 (0.924)**	-94.43	0.925 (0.923)	-94.80	0.927 (0.924)	-96.56	0.929 (0.925)
Abdomen	-15.92	0.875 (0.872)	-19.39	**0.876 (0.873)**	-18.93	0.878 (0.874)	-11.69	0.879 (0.873)
Tail	-86.87	0.739 (0.733)	-89.67	**0.741 (0.735)**	-81.39	0.742 (0.732)	-75.28	0.744 (0.732)
log(Claw)	-373.78	**0.918 (0.916)**	-374.73	0.918 (0.916)	-367.17	0.919 (0.916)	-364.47	0.920 (0.916)
Mineralisation								
log[Ca^{2+}]	201.30	**0.476 (0.421)**	202.01	0.472 (0.417)	209.73	0.483 (0.397)	216.15	0.501 (0.383)
log[Mg^{2+}]	166.35	**0.225 (0.143)**	164.97	0.235 (0.154)	172.14	0.255 (0.131)	176.70	0.292 (0.126)
log[Na^{+}]	150.72	**0.575 (0.529)**	152.26	0.568 (0.523)	156.84	0.590 (0.521)	161.83	0.609 (0.516)
log[K^{+}]	170.01	**0.564 (0.518)**	171.66	0.557 (0.510)	174.36	0.586 (0.518)	175.12	0.621 (0.532)
log[Sr^{+}]	208.42	**0.451 (0.393)**	209.07	0.448 (0.390)	216.90	0.459 (0.369)	223.14	0.478 (0.355)
[Mg^{2+}]:[Ca^{2+}]	-249.57	0.6502 (0.613)	-255.70	0.6701 (0.635)	-263.28	**0.7209 (0.674)**	-258.05	0.7333 (0.670)
Feeding rate	392.44	**0.133 (0.069)**	392.21	0.137 (0.073)	394.88	0.156 (0.059)		**0.308 (0.197)**
Resting metabolic rate (MO_2)	-751.02	0.746 (0.740)	-750.84	0.746 (0.740)	-756.62	**0.755 (0.746)**	-752.69	0.759 (0.746)

Table S5 ANOVA and post-hoc Tukey HSD results assessing the effects of ocean acidification (pCO_2 concentration groups: 1 – 400 µatm; 2 – 600 µatm; 3 – 800 µatm; 4 – 1000 µatm; 5 – 1200 µatm; 6 – 2000 µatm; 7 – 3000 µatm) in stage I larvae. Only significant metabolites are included in the table.

**Metabolite**	**Fvalue**	**p value**	**-log10(p)**	**FDR**	**Tukey's HSD**
C20.1	10.496	2.30E-07	6.6391	1.29E-05	2-1; 3-1; 5-1; 6-1; 7-1; 6-4; 7-4
C11.0	9.4508	8.37E-07	6.0774	2.34E-05	4-1; 5-1; 6-1; 7-1; 5-3; 6-3; 7-3
C17.1	8.8354	1.85E-06	5.7323	3.46E-05	2-1; 3-1; 4-1; 5-1; 6-1; 7-1
C20.5	7.4864	1.16E-05	4.9359	0.00016225	2-1; 3-1; 4-1; 5-1; 6-1; 7-1
Betaine	7.11	1.98E-05	4.7033	0.00019532	4-1; 5-1; 6-1; 7-1; 4-2; 5-2; 6-2; 7-2
Phospho.L.Arginine	7.0716	2.09E-05	4.6793	0.00019532	3-1; 4-1; 5-1; 6-1; 7-1; 6-2
C14.0	6.9389	2.54E-05	4.5959	0.00019974	2-1; 3-1; 4-1; 5-1; 6-1; 7-1
C22.6	6.8358	2.95E-05	4.5308	0.00019974	2-1; 3-1; 4-1; 5-1; 6-1; 7-1
C15.0	6.777	3.21E-05	4.4935	0.00019974	2-1; 3-1; 4-1; 5-1; 6-1; 7-1
C8.0	6.5098	4.76E-05	4.3223	0.00026659	2-1; 3-1; 4-1; 5-1; 6-1; 7-1
C20.4	5.7678	0.0001	3.8338	0.0007045	2-1; 3-1; 5-1; 6-1
C20.2	5.7011	0.0002	3.7889	0.0007045	2-1; 3-1; 4-1; 5-1; 6-1; 7-1
C15.1	5.6974	0.0002	3.7864	0.0007045	2-1; 4-1; 5-1; 6-1; 7-1; 5-3
Succinate	5.4579	0.0002	3.6238	0.00095127	3-1; 4-1; 5-1; 6-1; 5-2
ATP	5.34	0.0003	3.5429	0.0010269	3-1; 5-3; 6-3; 7-3
C18.3	5.3248	0.0003	3.5325	0.0010269	2-1; 3-1; 4-1; 6-1; 7-1
C10.0	4.8863	0.0006	3.2272	0.0019523	2-1; 3-1; 4-1; 5-1; 6-1; 7-1
C20.3	4.6966	0.0008	3.0929	0.0025121	2-1; 3-1; 5-1; 6-1; 7-1
C12.0	4.6195	0.0009	3.0379	0.0027011	3-1; 4-1; 5-1; 6-1; 7-1
C16.0	4.4369	0.0012	2.9069	0.0034693	4-1; 5-1; 5-3
a.ketoglutaric.acid	4.2124	0.0018	2.7442	0.0048054	2-1; 4-1
Valine	3.8722	0.0032	2.4944	0.0081539	5-3; 5-4; 6-5; 7-5
C18.1	3.8086	0.0036	2.4473	0.0086255	2-1; 3-1; 4-1; 5-1; 6-1; 7-1
NAD	3.7883	0.0037	2.4322	0.0086255	3-1; 6-1; 7-1
AMP	3.5234	0.0058	2.2344	0.013056	2-1; 4-1; 6-1; 7-1
C22.1	3.4618	0.0065	2.1881	0.013967	3-1; 4-1; 7-1
C18.2	3.4285	0.0069	2.163	0.014249	2-1; 6-1; 7-1
Malate	3.3963	0.0073	2.1388	0.01453	2-1; 3-1
C13.0	3.1207	0.0117	1.93	0.022686	2-1; 3-1; 4-1; 5-1; 7-1
8.Oxo.2.dexyguanosine	2.8749	0.0181	1.7426	0.033767	2-1; 3-1; 4-1; 5-1; 6-1; 7-1

Cystine	2.8282	0.0196	1.7068	0.035482	6-1; 7-1
a.Glycerophosphate	2.7281	0.0234	1.6302	0.04101	5-1; 7-1

Table S6. ANOVA and Tukey HSD post-hoc results assessing the effects of ocean acidification (*p*CO_2 concentration groups: 1 – 400 µatm; 2 – 600 µatm; 3 – 800 µatm; 4 – 1000 µatm; 5 – 1200 µatm; 6 – 2000 µatm; 7 – 3000 µatm) in stage II larvae. Only significant metabolites are included in the table.

**Metabolite**	**Fvalue**	**p value**	**-log10(p)**	**FDR**	**Tukey's HSD**
C20.5	8.993	3.5109E-6	5.4546	1.615E-4	7-1; 7-2; 7-3; 7-4; 7-5; 7-6
C18.1	6.4646	8.5935E-5	4.0658	0.0016617	7-1; 7-2; 7-3; 7-4; 7-5; 7-6
C20.4	6.2955	1.0837E-4	3.9651	0.0016617	7-1; 7-2; 7-3; 7-4; 7-5; 7-6
C18.2	5.9621	1.7242E-4	3.7634	0.0019828	7-1; 7-2; 7-3; 7-4; 7-5; 7-6
a.ketoglutaric.acid	5.5877	2.9386E-4	3.5319	0.002465	7-1; 7-2; 7-3; 7-4; 7-5; 7-6
C17.0	5.4259	3.7146E-4	3.4301	0.002465	7-1; 7-2; 7-3; 7-4; 7-5; 7-6
C16.1	5.4191	3.7511E-4	3.4258	0.002465	7-1; 7-2; 7-3; 7-4; 7-5; 7-6
Malate	5.1605	5.4833E-4	3.261	0.0030892	7-1; 7-2; 7-3; 7-4; 7-5; 7-6
C20.3	5.0851	6.1321E-4	3.2124	0.0030892	7-1; 7-3; 7-5; 7-6
C18.3	5.0241	6.7157E-4	3.1729	0.0030892	7-1; 7-2; 7-3; 7-4; 7-5; 7-6
Fumarate	4.2011	0.0023665	2.6259	0.0098963	7-2; 7-3; 7-5; 7-6
C20.2	4.0048	0.0032256	2.4914	0.012365	7-1; 7-2; 7-3; 7-4; 7-6
Alanine	3.8937	0.003849	2.4146	0.013088	5-1
Lactate	3.8723	0.0039832	2.3998	0.013088	5-3; 5-4
Sarcosine	3.3993	0.0085625	2.0674	0.026258	no significant pairwise comparisor
C22.6	3.303	0.010027	1.9988	0.028828	7-1; 7-2; 7-3; 7-4; 7-6
Phospho.L.Arginine	3.2648	0.010678	1.9715	0.028893	7-3
Methionine	2.9344	0.018472	1.7335	0.047207	3-1
a.Glycerophosphate	2.8748	0.020406	1.6902	0.049405	7-2; 7-5

Table S7: Physico-chemical parameters (mean ± SE) of the different pCO_2 treatments for larval setup ($n_{kreisels}$ = 4 per pCO_2 condition) and juvenile setup (n_{tanks} = 3 per pCO_2 condition). Seawater temperature, salinity, oxygen content, pH_T (pH on the total scale) and total alkalinity (A_t) were measured whereas the other parameters of the carbonate system were calculated with CO2sys software (Lewis & Wallace, 1998). pCO_2: CO_2 partial pressure; DIC: dissolved inorganic carbon; HCO_3^-: bicarbonates concentration; CO_3^{2-}: carbonates concentration; Ω_{Ca}: saturation state of seawater with respect to calcite and Ω_{Ar}: saturation state of seawater with respect to aragonite.

	“Larval” setup (28 kreisels) from 1st to 18th of July 2016						
	400 µatm	600 µatm	800 µatm	1000 µatm	1200 µatm	2000 µatm	3000 µatm
	n=59 except for salinity & A_T (n= 6)	n=4 except for salinity & A_T (n= 6)	n=68 except for salinity & A_T (n= 6)	n=68 except for salinity & A_T (n= 6)	n=68 except for salinity & A_T (n= 6)	n=56 except for salinity & A_T (n= 6)	n=51 except for salinity & A_T (n= 6)
Temperature (°C)	17.97 ± 0.03	17.95 ± 0.04	17.97 ± 0.03	17.95 ± 0.04	17.88 ± 0.05	17.96 ± 0.04	17.97 ± 0.03
Salinity	31.28 ± 0.03	31.37 ± 0.10	31.42 ± 0.08	31.40 ± 0.08	31.42 ± 0.06	31.37 ± 0.06	31.33 ± 0.06
Oxygen (% sat)	99.70 ± 1.04	96.89 ± 1.80	97.44 ± 1.66	98.17 ± 1.09	99.24 ± 1.03	97.98 ± 1.24	98.20 ± 1.22
pH_T	7.99 ± 0.00	7.85 ± 0.01	7.78 ± 0.01	7.72 ± 0.01	7.62 ± 0.01	7.38 ± 0.01	7.22 ± 0.01
A_T (µEq kg^{-1})	2098.27 ± 3.71	2097.75 ± 5.81	2100.07 ± 4.70	2092.04 ± 3.04	2098.65 ± 6.96	2107.73 ± 4.71	2100.10 ± 8.37
DIC (µmol kg^{-1})	1922.37 ± 1.18	1983.51 ± 4.03	2007.84 ± 3.34	2013.48 ± 3.67	2054.43 ± 4.11	2134.02 ± 3.56	2167.89 ± 4.76
pCO_2 (µatm)	437.35 ± 3.79	651.68 ± 21.51	778.03 ± 18.92	897.62 ± 33.96	1155.76 ± 36.47	2062.23 ± 63.16	2974.21 ± 75.12
HCO_3^- (µmol kg^{-1})	1773.58 ± 1.85	1858.46 ± 5.40	1892.84 ± 4.19	1903.57 ± 4.26	1950.58 ± 4.15	2023.85 ± 2.43	2037.57 ± 3.97
CO_3^{2-} (µmol kg^{-1})	133.54 ± 0.83	102.32 ± 2.21	87.87 ± 1.63	78.61 ± 1.70	63.47 ± 1.43	37.90 ± 0.95	26.59 ± 0.65
Ω_{Ca}	3.26 ± 0.02	2.50 ± 0.05	2.15 ± 0.04	1.92 ± 0.04	1.55 ± 0.03	0.93 ± 0.02	0.65 ± 0.02
Ω_{ar}	2.09 ± 0.01	1.60 ± 0.03	1.38 ± 0.03	1.23 ± 0.03	0.99 ± 0.02	0.59 ± 0.01	0.42 ± 0.01
	“Juvenile setup” (21 tanks) from 18th of July to 21st of August 2016						
	400 µatm	600 µatm	800 µatm	1000 µatm	1200 µatm	2000 µatm	3000 µatm
	n=105 except for salinity, A_T and DIC(n=6)	n=105 except for salinity & A_T (n= 6)	n=103 except for salinity & A_T (n= 6)	n=94 except for salinity & A_T (n= 6)	n=135 except for salinity & A_T (n= 6)	n=105 except for salinity & A_T (n= 6)	n=96 except for salinity & A_T (n= 6)
Temperature (°C)	17.92 ± 0.02	18.06 ± 0.13	18.10 ± 0.10	18.03 ± 0.12	17.98 ± 0.12	17.96 ± 0.01	17.96 ± 0.02
Salinity	31.47 ± 0.12	31.50 ± 0.12	31.47 ± 0.12	31.52 ± 0.08	31.52 ± 0.11	31.55 ± 0.06	31.58 ± 0.08
Oxygen (% sat)	98.62 ± 0.79	99.14 ± 1.12	99.41 ± 1.11	110.49 ± 11.06	99.15 ± 0.70	108.81 ± 9.39	98.45 ± 0.83
pH_T	7.97 ± 0.00	7.88 ± 0.01	7.79 ± 0.01	7.70 ± 0.01	7.66 ± 0.01	7.36 ± 0.01	7.14 ± 0.01
A_T (µmol kg^{-1})	2131.20 ± 5.33	2132.32 ± 3.67	2133.43 ± 4.52	2135.74 ± 6.16	2133.24 ± 4.43	2132.37 ± 4.98	2136.44 ± 5.15
DIC (µmol kg^{-1})	1959.24 ± 0.97	1992.08 ± 2.17	2024.90 ± 2.92	2054.68 ± 3.30	2067.09 ± 2.35	2154.74 ± 3.39	2231.76 ± 3.92
pCO_2 (µatm)	473.87 ± 2.61	601.79 ± 14.32	773.79 ± 25.52	968.56 ± 32.87	1061.44 ± 22.52	2201.55 ± 68.32	3714.80 ± 102.65
HCO_3^- (µmol kg^{-1})	1814.35 ± 1.16	1861.42 ± 2.98	1905.74 ± 3.75	1943.43 ± 4.12	1959.96 ± 2.94	2040.49 ± 2.02	2079.37 ± 1.28
CO_3^{2-} (µmol kg^{-1})	128.34 ± 0.59	109.84 ± 1.29	92.32 ± 1.67	77.91 ± 1.89	70.06 ± 1.32	37.43 ± 0.90	22.61 ± 0.50
Ω_{Ca}	3.13 ± 0.01	2.68 ± 0.03	2.26 ± 0.04	1.90 ± 0.05	1.71 ± 0.03	0.92 ± 0.02	0.55 ± 0.01
Ω_{ar}	2.01 ± 0.01	1.72 ± 0.02	1.45 ± 0.03	1.22 ± 0.03	1.09 ± 0.02	0.59 ± 0.01	0.35 ± 0.01

Figure S1. Metabolomic fingerprinting stages III to IV. Metabolome differences between pCO_2 groups in lobster larval stages III, IV and V **(a)** PCA 2D score plot with 95% confidence intervals for stage III, **(b)** PCA 2D score plot with 95% confidence intervals for stage IV, (c) **(c)** PCA 2D score plot with 95 % confidence intervals for stage V, **(d)** PLS-DA scores plot with 95% confidence intervals for stage V. Model validation was calculated by the prediction accuracy during training test statistic with 1000 permutations, p = 0.016, (e) PLS-DA Variable Importance in Projection scores (VIP > 1 for significance). None of the metabolites were significant in the univariate ANOVA (FDR 0.05). pCO_2 groups: 1 – 400 μatm, 2- 600 μatm, 3 – 800 μatm, 4 – 1000 μatm, 5 – 1200 μatm, 6 – 2000 μatm, 7 – 3000 μatm). Group 6 and 7 are lacking from stage V analysis due to mortality levels.

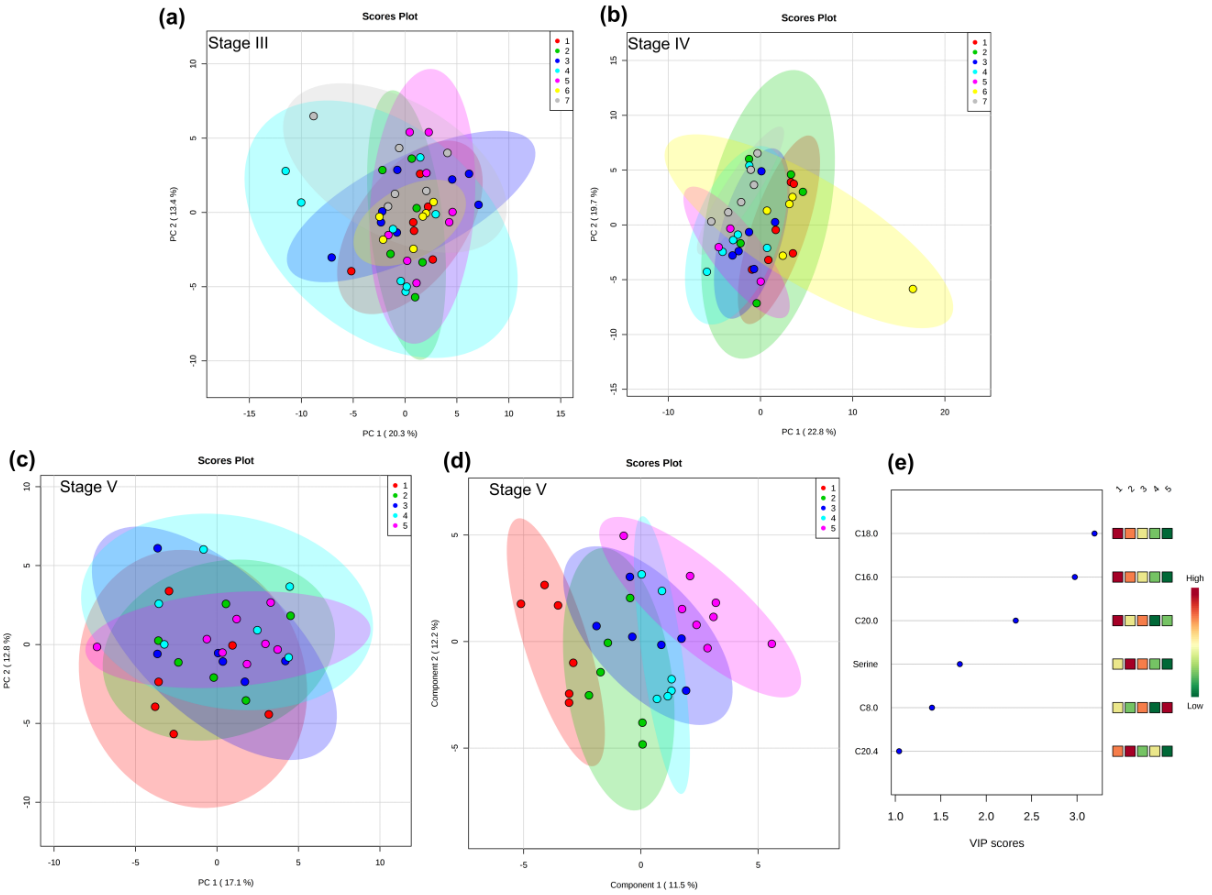

Supplement: Supplementary file 1 [file metabolites-11-00584-s001.zip › metabolites-1242513-supplementary.pdf]
